# Transcriptome profiling shows gene regulation patterns in a flavonoid pathway in response to exogenous phenylalanine in *Boesenbergia rotunda* cell culture

**DOI:** 10.1186/1471-2164-15-984

**Published:** 2014-11-18

**Authors:** Noor Diyana Md-Mustafa, Norzulaani Khalid, Huan Gao, Zhiyu Peng, Mohd Firdaus Alimin, Noraini Bujang, Wong Sher Ming, Yusmin Mohd-Yusuf, Jennifer A Harikrishna, Rofina Yasmin Othman

**Affiliations:** Centre for Research in Biotechnology for Agriculture (CEBAR), University of Malaya, 50603 Kuala Lumpur, Malaysia; Institute of Biological Sciences, Faculty of Science, University of Malaya, 50603 Kuala Lumpur, Malaysia; Centre for Foundation Studies in Science, University of Malaya, 50603 Kuala Lumpur, Malaysia; Centre of Research for Computational Sciences & Informatics in Biology, Bioindustry, Environment, Agriculture & Healthcare (CRYSTAL), University of Malaya, 50603 Kuala Lumpur, Malaysia; BGI-Guangzhou, No.280, Waihuan East Road, Guangzhou Higher Education Mega Center, Guangzhou, China; BGI-Shenzhen, Shenzhen, China

**Keywords:** *Boesenbergia rotunda*, panduratin A, anti-dengue, RNA-seq, differentially expressed genes (DEGs) analysis, phenylpropanoid pathway

## Abstract

**Background:**

Panduratin A extracted from *Boesenbergia rotunda* is a flavonoid reported to possess a range of medicinal indications which include anti-dengue, anti-HIV, anti-cancer, antioxidant and anti-inflammatory properties. *Boesenbergia rotunda* is a plant from the Zingiberaceae family commonly used as a food ingredient and traditional medicine in Southeast Asia and China. Reports on the health benefits of secondary metabolites extracted from *Boesenbergia rotunda* over the last few years has resulted in rising demands for panduratin A. However large scale extraction has been hindered by the naturally low abundance of the compound and limited knowledge of its biosynthetic pathway.

**Results:**

Transcriptome sequencing and digital gene expression (DGE) analysis of native and phenylalanine treated *Boesenbergia rotunda* cell suspension cultures were carried out to elucidate the key genes differentially expressed in the panduratin A biosynthetic pathway. Based on experiments that show increase in panduratin A production after 14 days post treatment with exogenous phenylalanine, an aromatic amino acid derived from the shikimic acid pathway, total RNA of untreated and 14 days post-phenylalanine treated cell suspension cultures were extracted and sequenced using next generation sequencing technology employing an Illumina-Solexa platform. The transcriptome data generated 101, 043 unigenes with 50, 932 (50.41%) successfully annotated in the public protein databases; including 49.93% (50, 447) in the non-redundant (NR) database, 34.63% (34, 989) in Swiss-Prot, 24,07% (24, 316) in Kyoto Encyclopedia of Genes and Genomes (KEGG) and 16.26% (16, 426) in Clusters of Orthologous Groups (COG). Through DGE analysis, we found that 14, 644 unigenes were up-regulated and 14, 379 unigenes down-regulated in response to exogenous phenylalanine treatment. In the phenylpropanoid pathway leading to the proposed panduratin A production, 2 up-regulated phenylalanine ammonia-lyase (PAL), 3 up-regulated 4-coumaroyl:coenzyme A ligase (4CL) and 1 up-regulated chalcone synthase (CHS) were found.

**Conclusions:**

This is the first report of *Boesenbergia rotunda de novo* transcriptome data that could serve as a reference for gene or enzyme functional studies in the Zingiberaceae family. Although enzymes that are directly involved in the panduratin A biosynthetic pathway were not completely elucidated, the data provides an overall picture of gene regulation patterns leading to panduratin A production.

**Electronic supplementary material:**

The online version of this article (doi:10.1186/1471-2164-15-984) contains supplementary material, which is available to authorized users.

## Background

*Boesenbergia rotunda* (Linnaeus) Mansfield, Kulturpflanze is a synonym of *Gastrochilus panduratum* Ridley, *Boesenbergia pandurata* (Roxb.), *Kaempferia pandurata* Roxb. and *Gastrochilus panduratus* (Roxb.) Ridl. and is believed to have originated from the Indian, Southern China and Southeast Asia regions [1–3]. It is a traditional medicinal plant known locally in Malaysia and Indonesia as temu kunci, merkunci, dekunci or temu kecil [3], in Thailand as kra-chai [4], in China as Chinese ginger or Chinese keys, while its English name is finger root ginger.

*Boesenbergia rotunda* (L.) is a perennial herb belonging to the Zingiberaceae family. It is a small herbaceous plant with short, slender rhizomes [5]. The rhizomes are widely used in Southeast Asia as an edible spice or vegetable and in ethnomedicine as an ingredient for the treatment of aphthous ulcers, dry mouth, stomach discomforts, leucorrhoea, dysentery, inflammation, rheumatism and muscular pains [3, 4]. Traditionally, their rhizomes are eaten raw to treat mouth ulcers [6] or prepared together with other medicinal plant rhizomes as a tonic for post-natal treatment to restore blood circulation and to rejuvenate the body [1, 6]. Crushed rhizomes are used externally to release stomach gas, improve appetite, improve digestion and treat rheumatism [1, 6].

The major bioactive constituents in *Boesenbergia rotunda* are flavonoids. To date, more than 20 flavonoids have been isolated from *Boesenbergia rotunda* and are classified into two main groups, flavanones and chalcones. Based on their flavonoid carbon skeleton structure, compounds that can be classified as flavanones include pinocembrin, pinostrobin, alpinetin, rotundaflavone I and rotundaflavone II, while cardamonin, 4-hydroxypanduratin A, panduratin A, isopanduratin A, boesenbergin A, krachaizin A and krachaizin B are classified as chalcones [7–12]. Among isolated secondary metabolites from *Boesenbergia rotunda*, panduratin A has been shown to possess various medicinal properties which include anti-dengue, anti-cancer, anti-inflammatory, anti-HIV-1 protease, antibacterial, anti-aging, antioxidant and anti-obesity properties [13–31].

Panduratin A and 4-hydroxypanduratin A were reported to exhibit stronger biological activities compared to other secondary metabolites in *Boesenbergia rotunda*[17]. In a previous study, panduratin A has been shown to have anti-dengue properties through inhibition of dengue-2 virus NS3 protease which eventually leads to the termination of viral replication [13]. Dengue is a fast emerging pandemic viral disease in tropical and sub-tropical regions worldwide [32]. The World Health Organization (WHO) reported that 2.5 billion people or about 40% of the world population, are now at risk of dengue with an estimated 50 – 100 million dengue infections worldwide annually [32]. Severe dengue or formally known as Dengue Haemorrhagic Fever has become leading cause of hospitalization and death. The WHO estimates that about 500 000 people are infected with severe dengue each year with 2.5% mortality [32]. To date there are no licensed dengue treatments while the frequency of dengue outbreaks are increasing each year [33].

Despite the extensive reports on the potential use of panduratin A, the limited amounts of panduratin A that can be extracted from their natural source has resulted in unmet market demands when up-scaled quantities are required. Harvesting of mature rhizomes require almost a one year planting cycle for *Boesenbergia rotunda*. In addition, extraction of panduratin A from 10 kilograms of dried *Boesenbergia rotunda* rhizome using a solvent extraction method only yields approximately 715.2 mg of panduratin A [21]. Although chemically synthesized panduratin A has been reported, the economics of the procedures continues to hinder large-scale production of panduratin A [34]. Alternatively the enhancement of panduratin A production through genetic manipulation of its secondary metabolic pathways is a potential strategy for panduratin A yield improvement and this would require knowledge of its biosynthetic pathway which at present remains unclear.

Panduratin A production has been shown in a published report from this laboratory to be enhanced by the addition of exogenous phenylalanine into *Boesenbergia rotunda* cell suspension cultures [35]. Phenylalanine is an aromatic amino acid produced from the shikimic acid pathway [36]. It provides the essential 6-carbon ring and 3-carbon side chain that is central to all phenylpropanoids. Phenylalanine is also the precursor for the production of cinnamic acid, the first phenylpropanoids in the phenylpropanoid pathway, which are eventually channelled into the production of most flavonoids in plants including panduratin A.

For elucidation of the genes that are involved in the panduratin A biosynthetic pathway, we have sequenced, and compared two sets of transcriptome profiles that were derived from phenylalanine treated and untreated (control) *Boesenbergia rotunda* cell suspension cultures. *De novo* transcriptome of *Boesenbergia rotunda* was done by combining both transcripts from control and treated samples to generate longer sequences. Subsequently, gene regulation patterns between the control and phenylalanine treated cell suspension cultures were analysed using DGE analysis by mapping both transcriptome profiles to the *de novo* transcriptome database. The focus of the research was to resolve the gene regulation patterns in the phenylpropanoid pathway that leads to panduratin A biosynthesis in *Boesenbergia rotunda* cell suspension cultures in response to exogenous phenylalanine. Additionally the *de novo* transcriptome data would also enrich the plant database and eventually serve as reference sequences for other Zingiberaceae family plant species.

## Results

### Short –read *de novo*sequencing and assembly

RNA samples were extracted from control and phenylalanine treated *Boesenbergia rotunda* callus using a modified CTAB method [37]. Illumina-Solexa RNA sequencing technology was used to sequence the whole transcriptome of *Boesenbergia rotunda*. After stringent data filtering and quality checks, approximately 50 million high-quality clean reads were obtained from both samples with 95.13% and 96.06% Q20 bases (base quality was more than 20) for control and treated sample respectively. In total, there were 24, 473, 594 and 23, 470, 0648 clean paired-end reads generated with a total of 3, 671, 039, 100 and 3, 520, 597, 200 nucleotides from control *Boesenbergia rotunda* callus and phenylalanine treated callus respectively (Table 1).

**Table 1:** **Summary of reads assembly generated by SOAPdenovo from control and phenylalanine treated** ***Boesenbergia rotunda*** **callus**

	Control	Phenylalanine treated
Total number of reads	24473594	23470648
Total nucleotides (nt)	3671039100	3520597200
GC%	49.31%	47.89%
Q20%	95.13%	96.06%
Step-wise assembly		
Total number of contig	287451	273979
Average sequence size of contigs	199	191
N50 length of contig	236	221
Total number of scaffolds	149648	147381
Average sequence size of scaffolds	359	330
N50 length of scaffold	535	465
Total number of unigenes	78998	77541
Total nucleotides (nt) in unigenes	44279890	39284596
Average sequence size of unigenes	561	507
N50 length of unigenes	703	610
Combined control and phenylalanine treated unigenes		
Total number of all unigenes	101043	
Average sequence size of all unigenes	599	
N50 length of all unigenes	804	
Unigenes with orientation	54284	
Unigenes without orientation	46759	

N50 size of contigs, scaffolds or unigene was calculated by ordering all sequences then adding the lengths from longest to shortest until the summed length exceeded 50% of the total length of all sequences.

Clean reads that were generated from the Illumina Genome analyzer were assembled into contigs, scaffolds and unigenes using open source SOAP denovo assembler program [38]. A total of 287, 451 and 273, 979 contigs with lengths ranging between 75 – 5680 bp and 75 – 3739 bp with N50 lengths of 236 and 221, for control and phenylalanine treated samples respectively. Contigs were then overlapped using paired-end read information to assemble into scaffolds. There were 149, 648 and 147, 381 scaffolds assembled from the control and treated samples with average scaffold sizes of 359 (control) and 330 (treated sample). Scaffolds from both samples lengths ranged from 100 to 12, 211 bp for the control and from 100 to 5, 943 bp for the treated sample.

Subsequently, scaffolds were overlapped and paired-end reads were used to fill the scaffold gaps to obtain unigenes. For the control sample, there were 78, 998 unigenes assembled with lengths ranging from 200 to 12, 209 bp and a N50 length of 703 bp; while for the treated sample, there were 77, 541 unigenes assembled with lengths ranging from 200 to 5, 944 bp with a N50 length of 610 bp. Finally, longer sequences denoted as All Unigenes, were assembled by overlapping both control and phenylalanine treated unigenes followed by removing redundant sequences using TGICL software. There were in total approximately 101, 043 All Unigenes assembled with lengths ranging from 200 to 12, 209 bp. The N50 lengths of All unigenes were 804 bp. Additional file 1 shows the length distribution, while Additional file 2 shows gap distribution of the control unigene, phenylalanine treated unigene and all unigenes respectively.

In order to determine the unigenes’ sequence orientation, all unigenes were aligned using BlastX alignment (*e* value < 1.00E -05) against four protein databases with the priority order of GenBank non-redundant (NR), Swiss-Prot, Kyoto Encyclopedia of Genes and Genomes (KEGG) and Clusters of Orthologous Groups (COG). Remaining unaligned unigenes were analyzed using ESTscan software [39] to predict the coding regions and to decide on sequence direction. The best-aligned results showed that 54, 284 unigenes are oriented while 46, 759 are non-oriented unigenes (Table 1).

### Functional annotation and gene ontology classification

Functional annotation gave information on protein function annotation, pathway annotation, COG annotation and Gene Ontology (GO) annotation. Unigenes with sequence orientation was aligned against public protein databases such as NR, Swiss-Prot, KEGG and COG using BlastX homology search (*e* value < 1.00E -05), which is based on sequence similarities to the published protein databases. There were in total 50, 932 (50.41%) unigenes successfully annotated (Table 2). Most of the unigenes were annotated using the NR database (49.93%) followed by Swiss-Prot (34.63%), KEGG (24.07%) and COG (16.26%). The remainder had no matches.

**Table 2:** **Functional annotation of** ***Boesenbergia rotunda*** **transcriptome data in four public protein databases**

Public protein database	No. of unigene hits	Percentage
NR	50447	49.93
Swiss-Prot	34989	34.63
KEGG	24316	24.07
COG	16426	16.26
Total	50932	50.41

The databases include Non-redundant (NR), Swiss-Prot, Kyoto Encyclopedia of Genes and Genomes (KEGG) and Cluster Orthologous Group (COG).

**Figure 1:**
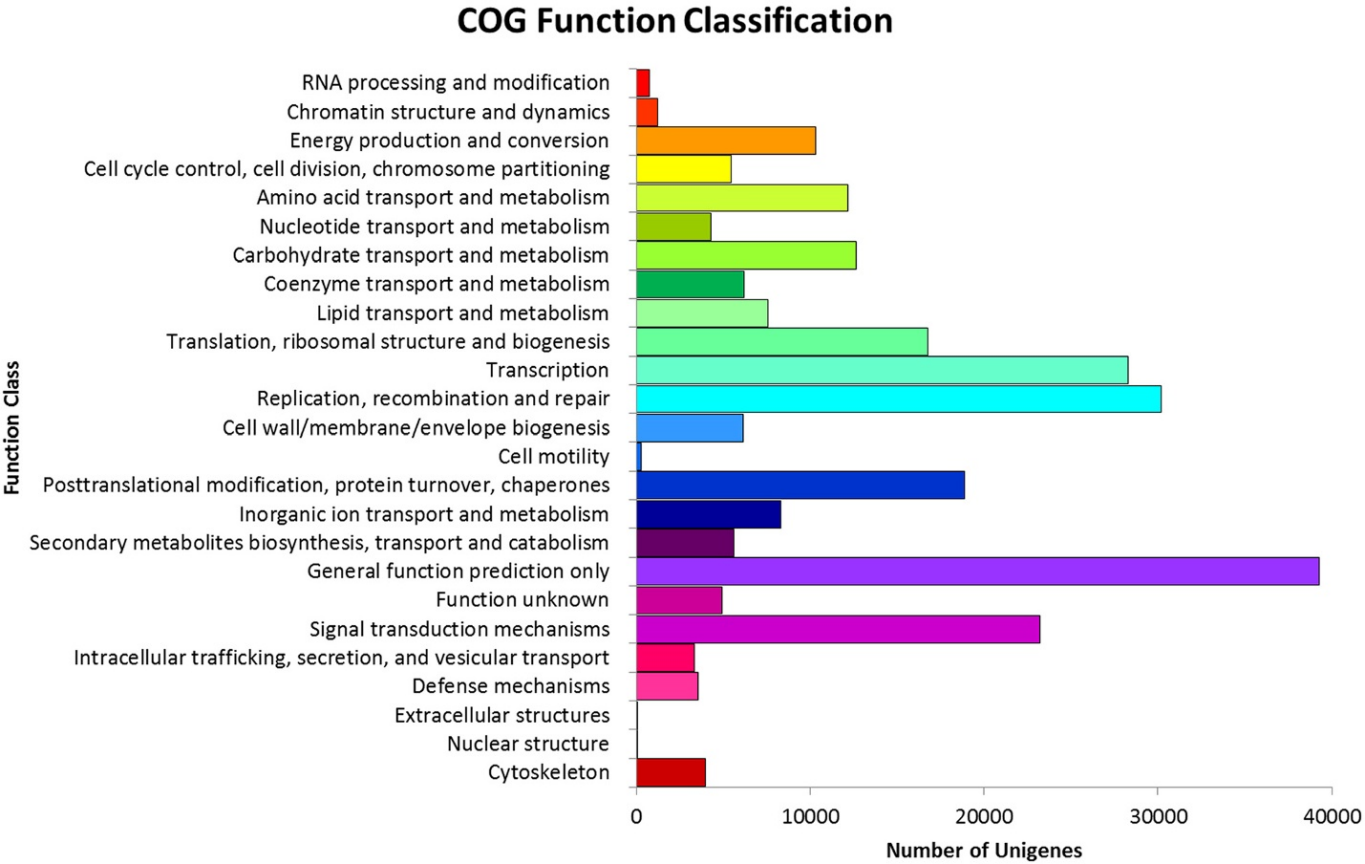
**Cluster Orthologous Group of protein functional annotation of** ***Boesenbergia rotunda*** **transcripts.**

Clusters Orthologous Groups of proteins (COG) database contain orthologous proteins that were classified under several categories. Unigenes were aligned to COG database to predict and classify their possible function. Figure 1 shows the distributions of 16, 526 unigenes assigned into 25 orthologous clusters in COG. Some unigenes may be assigned into several clusters in COG categories; while some unigenes were assigned to the same cluster but with different protein orthologous similarity. In total, there were 34, 434 unigenes that were assigned to COG database (Additional file 3). The majority of the unigenes were distributed in general function prediction (4, 851) followed by transcription (3, 691); and replication, recombination and repair (3, 053). A total of 1, 863 functionally unknown unigenes were identified. Whereas, 753 unigenes were assigned to secondary metabolites biosynthesis, transport and catabolism and 395 as defense mechanism unigenes.

**Figure 2:**
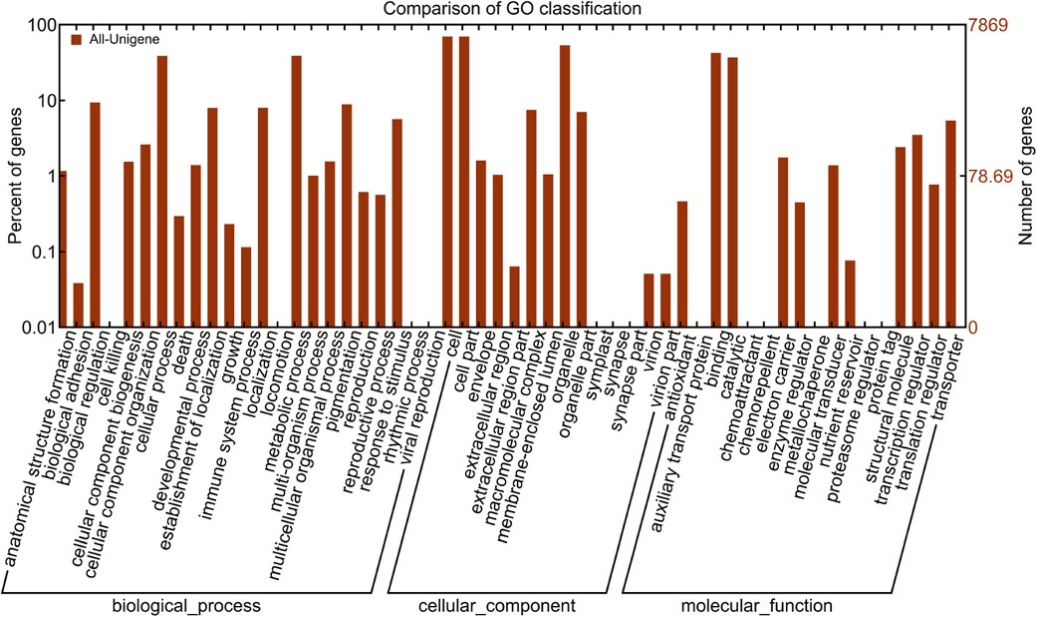
**Histogram presentation of unigene distributions in Gene Ontology (GO) functional classification.** Unigenes were further classified into sub-groups in biological process, cellular component and molecular function.

Unigene with NR annotation was further annotated and classified under Gene Ontology (GO). GO is an international standardized gene functional classification system. It has three ontologies which include molecular function, cellular component and biological properties. The basic unit of GO 0 s GO-term and every GO-term belongs to a type of ontology. Figure 2 shows the distribution of unigenes assigned in Gene Ontology. In total, there were 33,984 unigenes were mapped to GO with 7, 451 unigenes assigned to molecular function, 16,493 unigenes assigned to cellular components and 10, 040 unigenes assigned to biological process (Additional file 4). One unigene may be assign into several different GO-terms.

### Differentially expressed unigenes analysis

**Figure 3:**
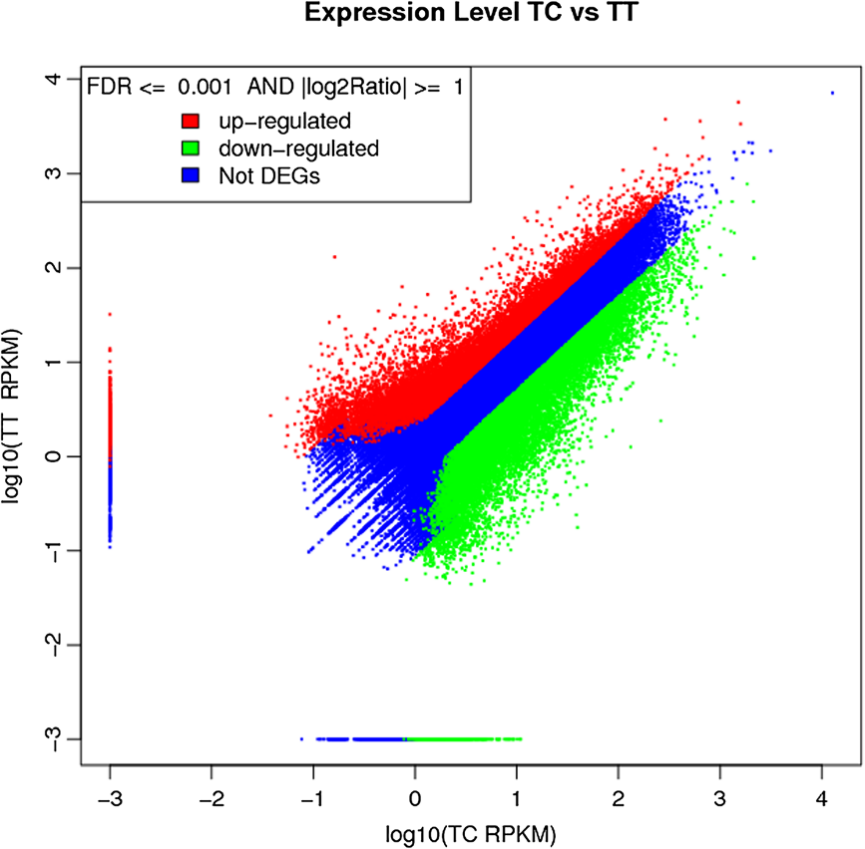
**Expression levels of differentially expressed genes in control (TC) and phenylalanine treated (TT) samples.** Up-regulated and down-regulated genes are denoted by red and green spots respectively, while not differentially expressed genes are denoted as blue spots.

Unigene expression was calculated using reads per kb per million reads (RPKM) method. Through this calculation, up-regulated and down-regulated of both control and phenylalanine treated transcripts were determined. However, in order to distinguish between significant and non-significant differentially expressed genes (DEGs), additional equations were employed. Significant differentially expressed genes (DEGs) were determined using Poisson distribution equation, with set the threshold of False Discovery Rate (FDR) lower or equal to 0.001 and the absolute value of log2 ratio lower or equal to 1 (Figure 3). In total, there were 14, 644 and 14, 379 unigenes showing significant differential expression respectively (Table 3).

**Table 3:** **Summary of differentially expressed genes (DEGs) expression levels in** ***Boesenbergia rotunda*** **transcriptome data**

	Total	Up	Down
Total DEGs	100869	47451	53418
Significant DEGs	29023	14644	14379
-with annotation	16018	6104	9914
-without annotation	13005	8540	4465

### Transcription factors and transcription regulators analysis

Transcription factors (TFs) and transcription regulators (TRs) play essential roles in regulating differentially expressed genes in both a spatial and temporal manner. In total, 139 transcription factors that are found in *Boesenbergia rotunda* can be further classified under 35 transcription factor families (Table 4). Based on the iTAK rice transcription factor database, 21 rice TFs were not found in *Boesenbergia rotunda*. The most abundant TFs found in *Boesenbergia rotunda* was C3H (17), followed by MYB (16), NAC (13), WRKY (9), bZIP (8) and AP2-EREBP (7). In response to phenylalanine treatment, eight TFs were up-regulated while twenty six up- TFs were down-regulated. Up-regulated TFs includes MYB, NAC, WRKY, bZIP, AP2-EREBP, G2-like, GRAS and C2C2-CO-like transcription factor.

**Table 4:** **Transcription factors identified in** ***Boesenbergia rotunda*** **based on transcription factors in the rice database using iTAK software**

	Transcription factor ( ***Boesenbergia rotunda*** )	Number of genes	Up-regulated	Down-regulated	Transcription factor ( ***Oryza sativa*** )	Number of genes
1	C3H	17	0	6	C3H	70
2	MYB	16	1	4	MYB	184
3	NAC	13	1	5	NAC	143
4	WRKY	9	1	2	WRKY	98
5	bZIP	8	1	1	bZIP	91
6	AP2-EREBP	7	1	2	AP2-EREBP	164
7	C2H2	5	0	0	C2H2	123
8	G2-like	5	1	1	G2-like	45
9	HB	5	0	1	HB	94
10	TUB	5	0	0	TUB	15
11	bHLH	4	0	0	bHLH	135
12	GRAS	4	1	0	GRAS	60
13	Tify	4	0	1	Tify	17
14	FAR1	3	0	0	FAR1	8
15	LOB	3	0	0	LOB	36
16	MADS	3	0	0	MADS	69
17	PBF-2-like	3	0	0	PBF-2-like	2
18	ABI3VP1	2	0	0	ABI3VP1	55
19	Alfin-like	2	0	0	Alfin-like	9
20	BBR/BPC	2	0	0	BBR/BPC	4
21	BSD	2	0	0	BSD	10
22	C2C2-GATA	2	0	0	C2C2-GATA	25
23	SRS	2	0	0	SRS	5
24	Trihelix	2	0	0	Trihelix	26
25	ARF	1	0	1	ARF	27
26	BES1	1	0	0	BES1	6
27	C2C2-CO-like	1	1	0	C2C2-CO-like	8
28	C2C2-YABBY	1	0	1	C2C2-YABBY	8
29	CCAAT	1	0	0	CCAAT	51
30	EIL	1	0	0	EIL	9
31	GeBP	1	0	0	GeBP	13
32	GRF	1	0	0	GRF	12
33	mTERF	1	0	1	mTERF	34
34	OFP	1	0	0	OFP	31
35	zf-HD	1	0	0	zf-HD	14
36	ARR-B	0	0	0	ARR-B	9
37	C2C2-Dof	0	0	0	C2C2-Dof	30
38	CAMTA	0	0	0	CAMTA	6
39	CPP	0	0	0	CPP	11
40	CSD	0	0	0	CSD	2
41	DB	0	0	0	DBP	3
42	E2F-DP	0	0	0	E2F-DP	8
43	FHA	0	0	0	FHA	18
44	HRT	0	0	0	HRT	1
45	HSF	0	0	0	HSF	25
46	LFY	0	0	0	LFY	2
47	LIM	0	0	0	LIM	6
48	PLATZ	0	0	0	PLATZ	15
49	RWP-RK	0	0	0	RWP-RK	13
50	S1Fa-like	0	0	0	S1Fa-like	2
51	SBP	0	0	0	SBP	19
52	Sigma70-like	0	0	0	Sigma70-like	6
53	TAZ	0	0	0	TAZ	6
54	TCP	0	0	0	TCP	21
55	ULT	0	0	0	ULT	2
56	VOZ	0	0	0	VOZ	2
Total	139	8	26		1908	

Subsequently, there were 46 transcription regulators which are classified under 15 families found in *Boesenbergia rotunda* (Table 5). The most abundant TRs found in *Boesenbergia rotunda* was orphan (9), followed by AUX/IAA (8) and SET (5). There were in total only 4 TRs that were up-regulated and 9 down-regulated in response to phenylalanine. Three up-regulated TRs were orphan, AUX/IAA and SET.

**Table 5:** **Transcription regulators identified in** ***Boesenbergia rotunda*** **based on transcription regulators in the rice database using iTAK software**

	Transcriptional regulator ( ***Boesenbergia rotunda*** )	Number of genes	Up-regulated	Down-regulated	Transcription regulator (Oryza sativa)	Number of genes
1	Orphans	9	2	3	Orphans	79
2	AUX/IAA	8	1	1	AUX/IAA	32
3	SET	5	1	0	SET	41
4	SNF2	4	0	2	SNF2	39
5	TRAF	4	0	1	TRAF	59
6	RB	3	0	0	RB	2
7	SWI/SNF-BAF60b	3	0	0	SWI/SNF-BAF60b	11
8	MED6	2	0	0	MED6	1
9	PHD	2	0	1	PHD	39
10	GNAT	1	0	0	GNAT	35
11	HMG	1	0	0	HMG	9
12	Jumonji	1	0	0	Jumonji	14
13	Rcd1-like	1	0	0	Rcd1-like	5
14	SOH1	1	0	0	SOH1	2
15	SWI/SNF-SWI3	1	0	1	SWI/SNF-SWI3	4
16	ARID	0	0	0	ARID	6
17	Coactivator p15	0	0	0	Coactivator p15	3
18	DDT	0	0	0	DDT	7
19	IWS1	0	0	0	IWS1	17
20	LUG	0	0	0	LUG	6
21	MBF1	0	0	0	MBF1	2
22	MED7	0	0	0	MED7	1
23	Pseudo ARR-B	0	0	0	Pseudo ARR-B	5
Total	46	4	9		419	

### Pathway analysis

**Figure 4:**
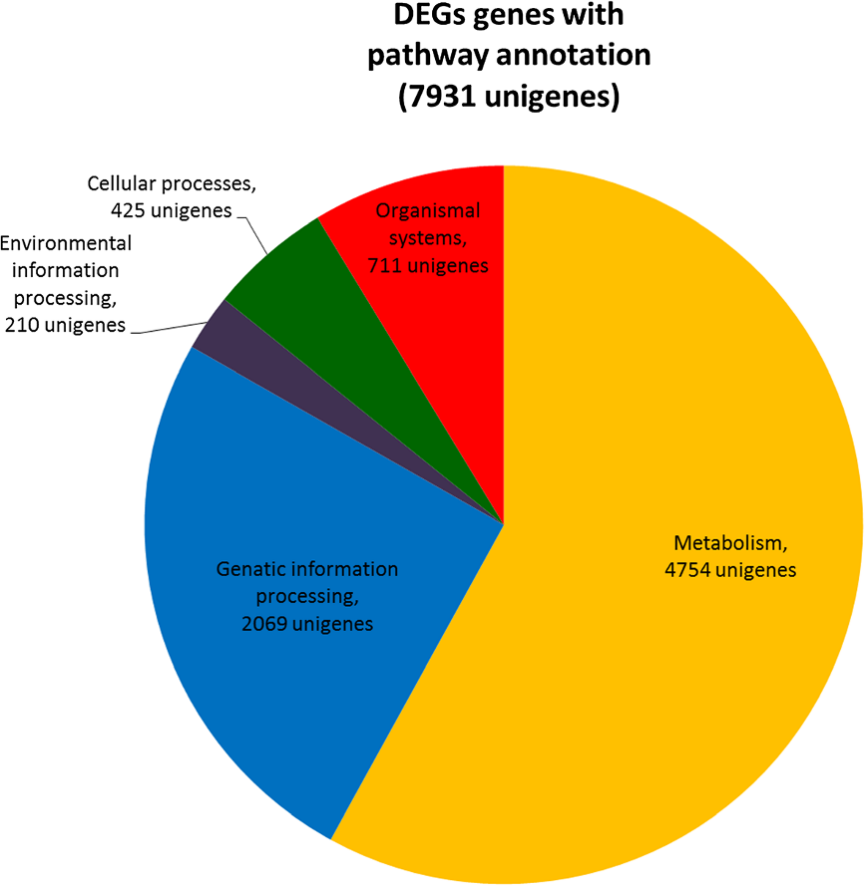
**Distribution chart of differentially expressed genes (DEGs) in KEGG.** Unigenes were distributed into five major KEGG categories; Metabolism, Genetic Information Processing, Environmental Information Processing, Cellular Processes and Organismal Systems.

**Figure 5:**
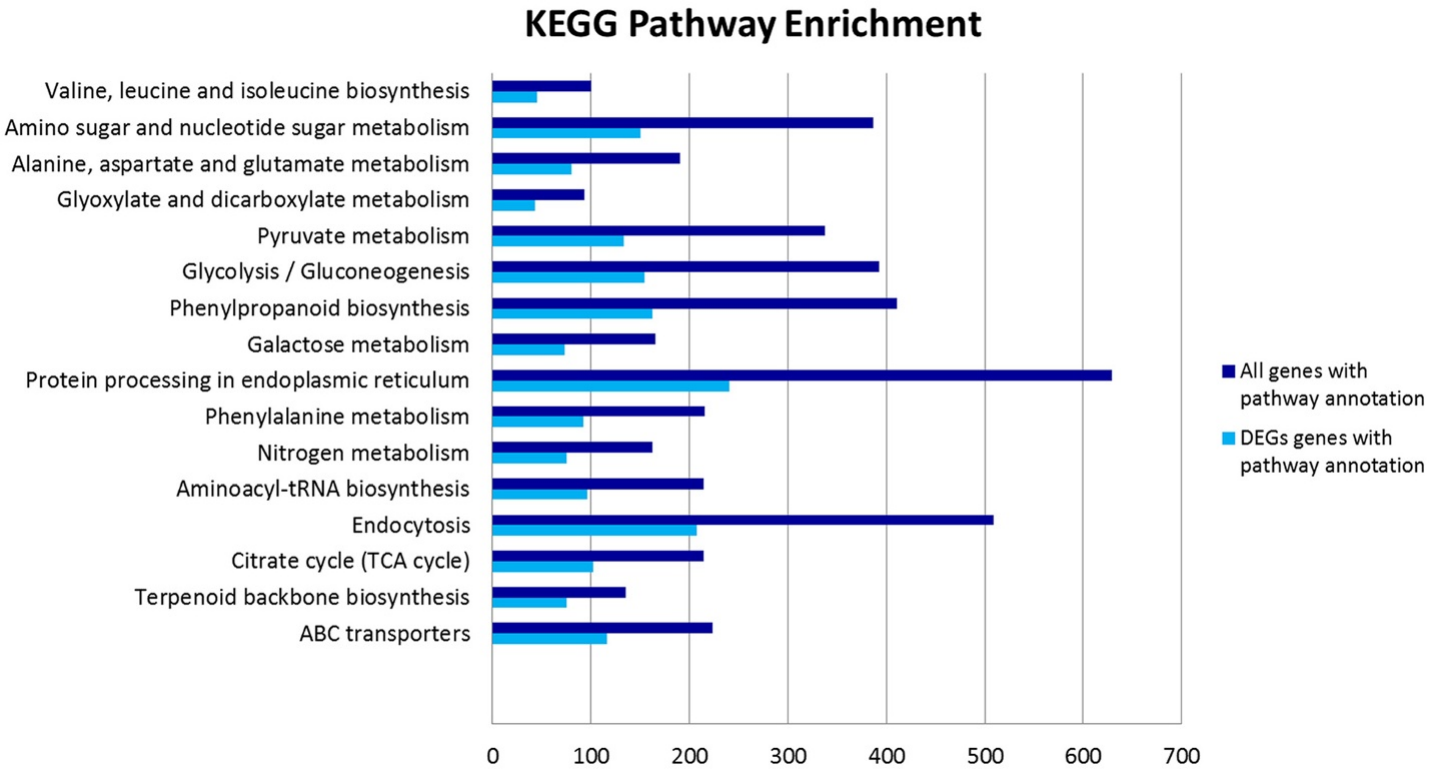
**Histogram of pathways that are enriched with differentially expressed genes.** The bar chart show the comparison between the number of all unigenes and differentially expressed genes that were mapped in sixteen KEGG pathways with Q value ≤ 0.05.

Pathway-based analysis provides information and further understanding on how *Boesenbergia rotunda* regulate their biological functions and synthesizes secondary metabolites in response to phenylalanine at the molecular level. Usually, unigenes in the same pathways cooperate with each other to exercise their biological functions. In total, there were 24, 316 unigenes that mapped to the KEGG plant database using BlastX homology search. These unigenes were classified under 166 KEGG pathways in five main categories in KEGG which includes Metabolism, Genetic Information Processing, Environmental Information Processing, Cellular Processes and Organismal Systems (Table 6). A single EC number may contain one or multiple unigenes. However, only 7,931 unigenes that were differentially expressed genes (DEGs), significantly up- or down-regulated, were mapped in the KEGG pathways. The total distribution of DEGs is represented in Figure 4. Out of 116 pathways, 16 pathways were significantly enriched with DEGs (Q value ≤ 0.05) (Table 7). Figure 5 shows the comparison between all unigenes and DEGs that mapped in DEG significantly enriched pathways.

**Table 6:** **Distributions of all unigenes and differentially expressed genes (DEGs) in KEGG database classification**

Category	Sub-category	All genes with pathway annotation	DEGs with pathway annotation
Metabolism	Amino Acid Metabolism	2139	736
	Biosynthesis of Other Secondary Metabolites	1092	385
	Carbohydrate Metabolism	3353	1297
	Energy Metabolism	937	352
	Glycan Biosynthesis and Metabolism	278	86
	Lipid Metabolism	1445	514
	Metabolism of Cofactors and Vitamins	503	143
	Metabolism of Other Amino Acids	581	213
	Metabolism of Terpenoid and Polyketides	622	235
	Nucleotide Metabolism	2290	721
	*Total*	*13240*	*4682*
Genetic Information Processing	Folding, Sorting and Degradation	2,062	729
	Replication and Repair	923	275
	Translation	3588	1137
	*Total*	*6,573*	*2,141*
Environmental Information Processing	Membrane Transport	224	116
	Signal Transduction	288	94
	*Total*	*512*	*210*
Cellular Processes	Transport and Catabolism	1251	452
	*Total*	*1251*	*452*
Organismal Systems	Environmental Adaptation	2122	675
	Immune System	140	36
	*Total*	*2262*	*711*
Total Unigenes		24316	7931

There are five major categories which include Metabolism, Genetic Information Processing, Environmental Information Processing, Cellular Processes and Organismal Systems.

**Table 7:** **Summary of unigene distribution in KEGG pathways that has significant differential expression of genes**

Category	Sub-category	Pathway	DEGs with pathway annotation	Up-regulated unigenes	Down-regulated unigenes	Q-value
Metabolism	Carbohydrate Metabolism	Citrate cycle (TCA cycle)	102	14	88	9.60E-05
		Galactose metabolism	73	18	55	1.43E-02
		Glycolysis/Gluconeogenesis	155	33	122	2.20E-02
		Amino sugar and nucleotide sugar metabolism	151	56	95	3.04E-02
		Pyruvate metabolism	134	18	116	3.04E-02
		Glyoxylate and dicarboxylate metabolism	43	7	36	3.04E-02
	Energy Metabolism	Nitrogen metabolism	75	25	50	3.56E-03
	Amino Acid Metabolism	Phenylalanine metabolism	92	51	41	1.43E-02
		Alanine, aspartate and glutamate metabolism	80	20	60	3.04E-02
		Valine, leucine and isoleucine biosynthesis	45	7	38	4.21E-02
	Metabolism of Terpenoid and Polyketides	Terpenoid backbone biosynthesis	75	4	71	1.90E-06
	Biosynthesis of Other Secondary Metabolites	Phenylpropanoid biosynthesis	163	68	95	1.43E-02
Genetic Information Processing	Translation	Aminoacyl-tRNA biosynthesis	96	10	86	2.44E-03
	Folding, Sorting and Degradation	Protein processing in endoplasmic reticulum	241	53	188	1.43E-02
Environmental Information Processing	Membrane Transport	ABC transporters	116	17	99	1.22E-07
Cellular Processes	Transport and Catabolism	Endocytosis	208	44	164	9.54E-04

Pathways in KEGG that has significant differential expression of genes was determined by Q value ≤ 0.05.

The most abundant DEGs were classified in the Metabolism category with 4,754 unigenes (Table 6), followed by Genetic Information Processing (2, 069 unigenes), Organismal Systems (711 unigenes), Cellular Processes (452 unigenes) and Environmental Information Processing (210 unigenes). There were ten sub-categories found under the Metabolism category (Table 6). In the Metabolism category, most of the DEGs were found in the carbohydrate metabolism sub-category (38.68%), followed by metabolism of terpenoid and polyketides (37.78%), metabolism of other amino acid (36.66%), energy metabolism (35.57%) and biosynthesis of other secondary metabolites (35.26%). Unigenes that were annotated in the Metabolism category were involved in functions related to catalysis of metabolism processes or generation of energy for primary and secondary metabolite production.

**Figure 6:**
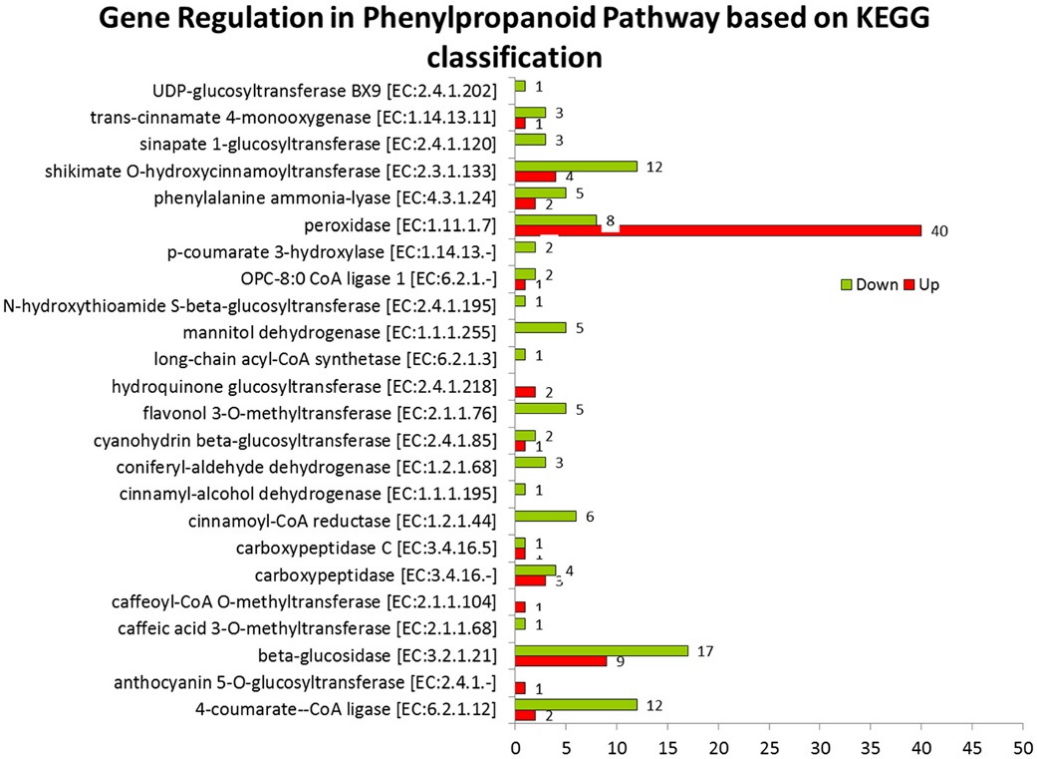
**Histogram of gene regulation in phenylpropanoid pathway based on KEGG database classification.**

Under the carbohydrate metabolism sub-category, there were six pathways that had a calculated Q value ≤ 0.05 (Table 7). The pathway in this sub-category with the smallest Q value was the citrate cycle pathway with 102 DEGs, followed by galactose metabolism (73 DEGs), glycolysis/gluconeogenesis (155 DEGs), amino sugar and nueleotide sugar metabolism (151 DEGs), pyruvate metabolism (134 DEGs) and glyoxylate and dicarboxylate metabolism (43 DEGs). Subsequently, for the energy metabolism sub-category, there was only one pathway identified which was for nitrogen metabolism with 75 DEGs. There were three pathways with Q value lower than 0.05 in the amino acid metabolism sub-category, which included phenylalanine metabolism (92 DEGs), alanine, aspartate and glutamate metabolism (80 DEGs) and valine, leucine and isoleucine biosynthesis (45 DEGs). Two secondary metabolite pathways with Q values lower than 0.05 were identified as terpenoid backbone biosynthesis (75 DEGs) and phenylpropanoid biosynthesis (163 DEGs). Based on the histogram in Figure 6, there were 68 unigenes up-regulated and 95 unigenes down-regulated. The highest number of up-regulated unigenes was peroxidase with 40 unigenes, while the highest down-regulated unigene was beta-glucosidase with 17 unigenes.

The Genetic Information Processing category consists of four sub-categories which include transcription (270 DEGs), translation (184 DEGs), folding, sorting and degradation (729 DEGs) and replication and repair (275 DEGs) (Table 6). Unigenes in this category mainly function in processing the correct transcription and translation processes. Aminoacyl-tRNA biosynthesis (96 DEGs) and protein processing in endoplasmic reticulum (241 DEGs) pathways in this category were shown to have Q value lower than 0.05 (Table 7).

Environmental Information Processing category consists of two subcategories which includes membrane transport (116 DEGs) and signal transduction (94 DEGs) (Table 6). However, only ABC transporter pathway (116 DEGs) under membrane transport was categorised with a Q value lower than 0.05 (Table 7). In plants, there is only one sub-category in the Cellular Processes category. Transport and catabolism sub-category consists of 452 DEGs (Table 6) with 208 DEGs in endocytosis pathway showing Q values lower than 0.05 (Table 7). Finally, environmental adaptation (675 DEGs) and immune systems (36 DEGs) were classified under the Organismal Systems category (Table 6). None of the pathways in this category had Q values lower than 0.05. Table 7 also shows gene regulation patterns in the selected KEGG pathways. There are more down-regulated unigenes than up-regulated unigenes in all the pathways with the exception of the phenylalanine metabolism pathway which had more unigenes being up-regulated (Table 7).

### Representation of genes regulation in phenylpropanoid pathway and flavonoid pathway

In the transcriptome data, we found that 411 unique unigenes were mapped to the phenylpropanoid pathway while 211 unigenes were mapped to the flavonoid pathway. In the phenylpropanoid pathway, 68 unigenes were up-regulated while 95 unigenes were down-regulated. Whereas in the flavonoid pathway, 11 unigenes were up-regulated and 42 unigenes were down-regulated. One unigene may map to more than one enzyme in the pathway (Tables 8 and 9). Tables 8 and 9 shows the unigenes that might be involved in panduratin A biosynthesis and the number of up- and down-regulated unigenes with their respective gene regulation patterns (Figure 6). Additional files 5 and 6 show the gene regulation patterns in phenylpropanoid and flavonoid pathway, respectively. The most abundant unigenes that were mapped to the phenylpropanoid pathway was peroxidases (EC: 1.11.1.7) with a total of 90 unigenes. There were 40 unigenes that showed up-regulation while only 8 unigenes were down-regulated (Table 8).

**Table 8:** **Unigenes potentially related to panduratin A biosynthesis in phenylpropanoid pathway**

Enzyme name	Abbreviations in the flavanoid pathway (Figure 7 )	EC number	Enzyme class	Total unigene	Up- regulated	Down-regulated
*Phenylpropanoid pathway*						
cinnamyl-alcohol dehydrogenase	-	1.1.1.195	Oxidoreductase	10	0	6
peroxidase	-	1.11.1.7	Oxidoreductase	90	40	8
ferulate-5-hydroxylase	-	1.14.-.-	Oxidoreductase	4	0	0
p-coumarate 3-hydroxylase	-	1.14.13.-	Oxidoreductase	5	0	2
trans-cinnamate 4-monooxygenase	C4H	1.14.13.11	Oxidoreductase	14	1	3
cinnamoyl-CoA reductase	-	1.2.1.44	Oxidoreductase	18	0	6
coniferyl-aldehyde dehydrogenase	-	1.2.1.68	Oxidoreductase	6	0	3
putative caffeoyl-CoA 3-O-methyltransferase	-	2.1.1.-	Transferase	2	0	0
caffeoyl-CoA O-methyltransferase	-	2.1.1.104	Transferase	2	1	0
caffeic acid 3-O-methyltransferase	-	2.1.1.68	Transferase	14	0	6
shikimate O-hydroxycinnamoyltransferase	-	2.3.1.133	Transferase	43	4	12
sinapoylglucose-choline O-sinapoyltransferase	-	2.3.1.91	Transferase	13	0	0
sinapoylglucose-malate O-sinapoyltransferase	-	2.3.1.92	Transferase	7	0	0
coniferyl-alcohol glucosyltransferase	-	2.4.1.111	Transferase	24	0	0
sinapate 1-glucosyltransferase	-	2.4.1.120	Transferase	29	1	5
beta-glucosidase	-	3.2.1.21	Hydrolase	76	9	17
phenylalanine ammonia-lyase	PAL	4.3.1.24	Lyase	14	2	5
phenylalanine/tyrosine ammonia-lyase	-	4.3.1.25	Lyase	1	0	0
4-coumarate--CoA ligase	4CL	6.2.1.12	Ligase	44	3	15

One unigene may map to more than one enzyme in the pathway. The Table shows all unigenes that are mapped to specific enzymes and gene regulation patterns either up-, down-regulated or both.

**Table 9:** **Unigenes potentially related to panduratin A biosynthesis in the flavonoid pathway**

Enzyme name	Abbreviations in flavanoid pathway (Figure 7 )	EC number	Enzyme class	Total unigene	Up-regulated	Down-regulated
*Flavonoid pathway*						
bifunctional dihydroflavonol 4-reductase/flavanone 4-reductase	DFR	1.1.1.219/ 1.1.1.234	Oxidoreductase	15	0	4
leucoanthocyanidin dioxygenase/ anthocyanin synthase	ANS	1.14.11.19	Oxidoreductase	16	3	4
flavone synthase	FS1/FS2	1.14.11.22	Oxidoreductase	0	0	0
flavonol synthase	FLS	1.14.11.23	Oxidoreductase	29	5	6
naringenin 3-dioxygenase/flavanone-3-hydroxylase	F3H	1.14.11.9	Oxidoreductase	20	2	5
p-coumarate 3-hydroxylase	-	1.14.13.-	Oxidoreductase	5	0	2
trans-cinnamate 4-monooxygenase	C4H	1.14.13.11	Oxidoreductase	14	1	3
flavonoid 3′-monooxygenase	-	1.14.13.21	Oxidoreductase	15	0	5
cytochrome P450, family 75, subfamily A (flavonoid 3′,5′-hydroxylase)	-	1.14.13.88	Oxidoreductase	6	0	2
leucoanthocyanidin reductase	LAR	1.17.1.3	Oxidoreductase	4	0	1
anthocyanin reductase	ANR	1.3.1.77	Oxidoreductase	0	0	0
caffeoyl-CoA O-methyltransferase	-	2.1.1.104	Transferase	2	1	0
shikimate O-hydroxycinnamoyltransferase	-	2.3.1.133	Transferase	43	4	12
6′-deoxychalcone synthase	-	2.3.1.170	Transferase	15	0	0
chalcone synthase	CHS	2.3.1.74	Transferase	25	1	7
chalcone isomerase	CHI	5.5.1.6	Isomerase	2	0	0

One unigene may map to more than one enzyme in the pathway. The table shows the all unigenes that are mapped to specific enzymes and gene regulation patterns either up-, down-regulated or both.

**Figure 7:**
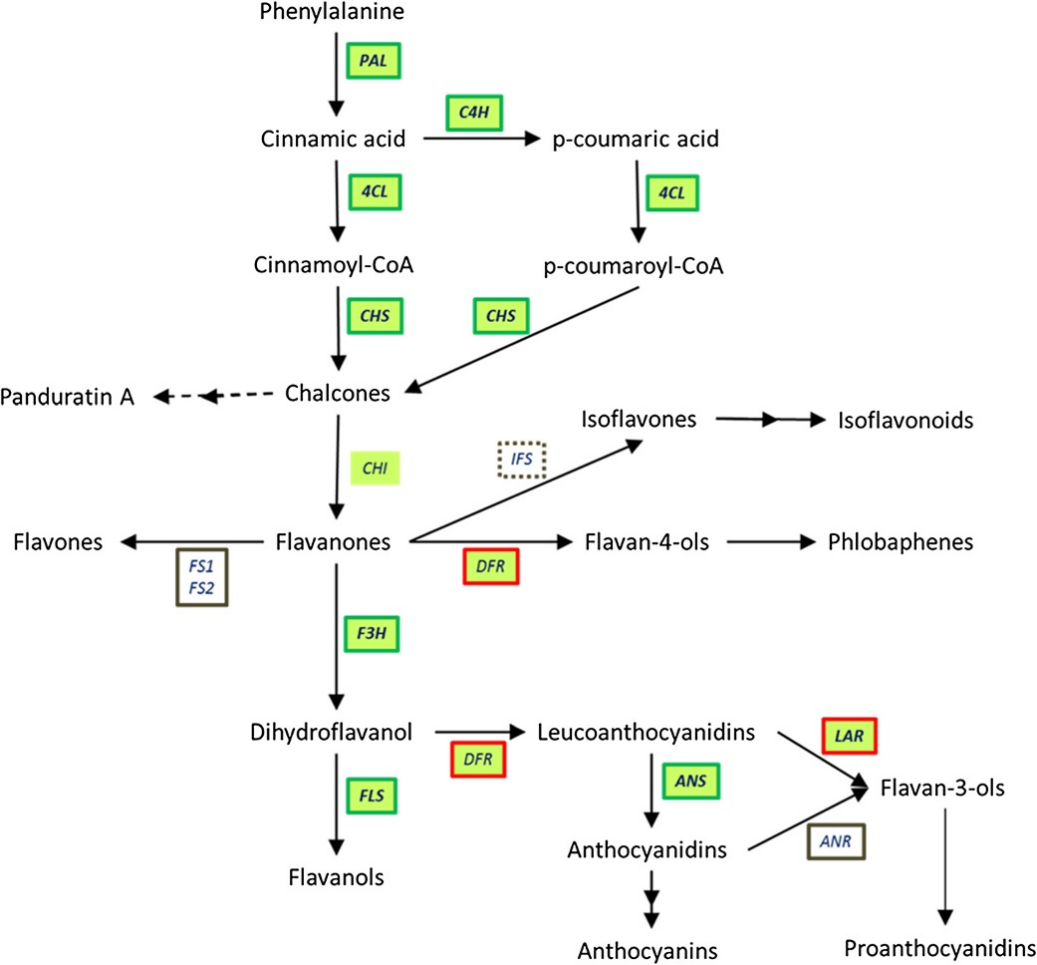
**General flavonoid biosynthetic pathway adapted from Bowsher,** ***et*** **.** ***al*** **., 2008** [72]**.** The pathway showing synthesis of major flavonoid groups which include chalcones, flavanones, flavones, flavan-4-ols, flavan-3-ols, flavanols, isoflavones and anthocyanins. Panduratin A, a chalcone-derived compound was proposed to derive from pinocembrin chalcone with dotted arrows. Abbreviations: PAL, phenylalanine ammonia lyase; C4H, cinnamate-4-hydroxylase; 4CL, 4-coumaroyl:coenzyme A ligase; CHS, chalcone synthase; CHI, chalcone isomerase; FS1/FS2, flavone synthase 1 and 2; IFS, isoflavone synthase; DFR, dihydroflavonol 4-reductase; F3H, flavanone-3-hydroxylase; FLS, flavonol synthase; ANS, anthocyanidin synthase; LAR, leucoanthocyanidin reductase and ANR, anthocyanidin reductase. Green box indicate that the unigenes mapped to the corresponding enzymes. The green boxes with green border consist of both up- and down-regulated unigene while green box with red border indicate down-regulated unigene. Brown border indicate that there were no unigene mapped to the corresponding enzymes, whereas the dotted borders indicate that the enzyme or the entire isoflavonoid pathway does not exist in *Boesenbergia rotunda*.

Panduratin A is a chalcone derivative that is proposed to be derived from the flavonoid pathway (Figure 7). Phenylalanine is an aromatic amino acid that is produced in the shikimic acid pathway [36] and enters the phenylpropanoid pathway as an initial substrate to produce all phenylpropanoids including flavonoids (Additional file 5). It provides the essential 6-carbon ring and 3-carbon side chain that is central to all phenylpropanoids and subsequently enters the flavonoid pathway to produce major flavonoid groups such as chalcones, flavanones, flavones, flavan-4-ols, flavan-3-ols, flavanols, isoflavones and anthocyanins (Additional file 6 and Figure 7).

An overview of the flavonoid biosynthetic pathway leading to the synthesis of major flavonoid groups and the proposed panduratin A biosynthesis is shown in Figure 7. Initially, phenylalanine is deaminated by phenylalanine ammonia lyase (PAL; EC: 4.3.1.24) to produce first the phenylpropanoid acid, cinnamic acid. Then, cinnamic acid was either converted to cinnamoyl-CoA by 4-coumaroyl:coenzyme A ligase (4CL; EC: 6.2.1.12) or converted to p-coumaric acid by a P450 cytochrome monoxygenase enzyme, cinnamate-4-hydroxylase (C4H; EC: 1.14.13.11). This was the first branching in the phenylpropanoid pathway. Subsequently, p-coumaric acid is also converted to phenolic CoA thioesters catalyzed by 4CL through attachment of CoA to a phenolic compound, producing p-coumaroyl-CoA.

Both phenolic CoA thioesters enter the flavonoid pathway and produce chalcones, which is the first flavonoid major group, by condensation of three acetate extender molecules from malonyl-CoA. The enzyme that is responsible for this reaction is chalcone synthase (CHS; EC: 2.3.1.74), a type III polyketide synthase [40]. Once produced, chalcones serve as the precursors for all of the various groups of flavonoids. Next, chalcone isomerase (CHI; EC: 5.5.1.6) converts chalcone into flavanone by isomerization. Flavanones are important intermediates, as they are involved in producing several other major flavonoid groups.

There are four major ways of producing various flavonoid groups by modifications of flavanones. Firstly, flavanones may be dehydrated to produce flavones by flavone synthase 1 (FS1; EC: 1.14.11.22) and 2 (FS2; EC: 1.14.11.22). Secondly, flavanones may also be further isomerized to form isoflavones by isoflavone synthase (ISF; EC: 1.14.13.136) in some plants, which are subsequently used to synthesize isoflavonoids. Thirdly, reduction reactions catalyzed by dihydroflavonol 4-reductase (DFR; EC: 1.1.1.219/1.1.1.234) converting flavanones to flavan-4-ols, which serves as precursors to produce phlobaphene polymers. Lastly, flavanones may form dihydroflavonols by hydroxylation catalyzed by flavanone-3-hydroxylase (F3H; EC: 1.14.11.9). Dihydroflavonols are further converted to flavanols by a desaturation reaction catalyzed by flavonol synthase (FLS; EC: 1.14.11.23).

Dihydroflavonols are precursors for anthocyanin pigments synthesis which upon synthesis is reduced to leucoanthocyanidins by dihydroflavonol 4-reductase (DFR) and converted to anthocyanidins by anthocyanidin synthase (ANS; EC: 1.14.11.19). Finally, anthocyanins are synthesized from anthocyanidins through further modifications. Leucoanthocyanidins and anthocyanidins are reduced to form flavan-3-ols by leucoanthocyanidin reductase (LAR; EC: 1.17.1.3) and anthocyanidin reductase (ANR; EC: 1.3.1.77) respectively which then serve as polymers producing proanthocyanidins.

Figure 7 shows the regulation patterns of unigenes that were mapped to the main enzymes in the flavonoid pathway. Only PAL, C4H, 4CL, CHS, CHI, F3H, FLS, DFR, ANS and LAR were mapped to the *Boesenbergia rotunda* transcriptome unigenes. However, no gene regulation was detected for CHI (Table 10). In contrast, no unigene matched for FS1/FS2 and ANR (Table 9). Additionally, the isoflavone biosynthetic pathway map was not present in the *Boesenbergia rotunda* system. In total, there were 14 unigenes mapped to PAL but only 2 of them showed up-regulation while 5 were down-regulated (Table 8). Out of 14 unigenes assigned as C4H, only 1 unigene was up-regulated and the other 3 unigenes were down-regulated. The most abundant unigene was assigned as 4CL with 44 unigenes. However, only 3 were up-regulated, while 15 others were down-regulated. A further 25 unigenes were assigned as CHS but only one unigene was up-regulated while 7 were down-regulated during production of chalcones in the flavonoid pathway. There was no gene regulation pattern observed during flavanone production from chalcones by CHI. A non-enzymatic reaction is suggested to be involved in this step. Subsequently, only two F3H and five FLS unigenes were up-regulated to form flavonols from flavanones. In contrast, there were four down-regulated DFR, three up-regulated ANS and one down-regulated LAR unigenes involved in anthocyanin and proanthocyanidin production.

**Table 10:** **Gene regulation patterns in the flavonoid pathway**

Enzyme	EC number	Up-regulated		Down-regulated	
		Unigene ID	Expression level fold	Unigene ID	Expression level fold
PAL	4.3.1.24	Unigene10327_All	1.1	Unigene83336_All	−1.9
		Unigene89418_All	1	Unigene56631_All	−1.9
				Unigene64872_All	−1.6
				Unigene619_All	−1.3
				Unigene9322_All	−1
C4H	1.14.13.11	Unigene67845_All	1.4	Unigene17324_All	−2.5
				Unigene11543_All	−1.7
				Unigene93243_All	−1.1
4CL	6.2.1.12	Unigene41852_All	2.2	Unigene88072_All	−3.3
		Unigene36813_All	1.2	Unigene37844_All	−3
		Unigene3277_All	1.1	Unigene68813_All	−2.8
				Unigene44539_All	−2.3
				Unigene51006_All	−2.2
				Unigene32973_All	−2.1
				Unigene85725_All	−2
				Unigene28297_All	−1.9
				Unigene57823_All	−1.8
				Unigene520_All	−1.7
				Unigene19555_All	−1.7
				Unigene20812_All	−1.5
				Unigene10021_All	−1.4
				Unigene6803_All	−1.4
				Unigene20574_All	−1.4
CHS	2.3.1.74	Unigene1735_All	1.5	Unigene35484_All	−2.2
				Unigene31906_All	−1.8
				Unigene37184_All	−1.4
				Unigene33635_All	−1.3
				Unigene63145_All	−1.3
				Unigene55042_All	−1.1
				Unigene29406_All	−1.1
F3H	1.14.11.9	Unigene49558_All	3.8	Unigene100816_All	−1.6
		Unigene5973_All	1.4	Unigene4657_All	−1.6
				Unigene22973_All	−1.4
				Unigene4884_All	−1.3
				Unigene23932_All	−1.1
FLS	1.14.11.23	Unigene49558_All	3.8	Unigene100816_All	−1.6
		Unigene89505_All	1.8	Unigene4657_All	−1.6
		Unigene56837_All	1.8	Unigene22973_All	−1.4
		Unigene26406_All	1.6	Unigene4884_All	−1.3
		Unigene5973_All	1.4	Unigene23932_All	−1.1
				Unigene33774_All	−1
DFR	1.1.1.219			Unigene100192_All	−2.8
				Unigene40110_All	−1.9
				Unigene84008_All	−1.7
				Unigene49734_All	−1.3
ANS	1.14.11.19	Unigene49558_All	3.8	Nigene100816_All	−1.6
		Unigene89505_All	1.8	Unigene4657_All	−1.6
		Unigene56837_All	1.8	Unigene4884_All	−1.3
				Unigene30270_All	−1.1
LAR	1.17.1.3			Unigene73982_All	−1

The genes include phenylalanine ammonia lyase (PAL), cinnamate-4-hydroxylase (C4H), 4-coumarate-CoA ligase (4CL), chalcone synthase (CHS), favanone-3-hydroxylase (F3H), flavonol synthase (FLS), dihydroflavonol-4-reductase (DFR), anthocyanin synthase (ANS) and leucoanthocyanidin reductase (LAR).

Based on gene regulation patterns in the flavonoid biosynthetic pathway, the highest up-regulated expression level was Unigene49558_All with 3.8 fold higher compared to control (Table 10). This unigene was annotated as F3H, FLSand ANS. The second highest up-regulated gene was Unigene41852_All, which annotated as 4CL which showed a 2.2 fold change. The rest of the unigenes in the flavonoid pathway were 1 to 1.8 fold up-regulated. The most down-regulated expression in the flavonoid pathway was 4CL with seven out of fifteen unigenes showing expression levels between 2 to 3.3 fold lower than the control (Table 10). The unigenes included Unigene88072_All, Unigene37844_All, Unigene68813_All, Unigene44539_All, Unigene51006_All, Unigene32973_All and Unigene85725_All. Three other unigenes that showed more than 2 fold down-regulation was C4H (Unigene17324_All) with 2.5 fold, CHS (Unigene35484_All) with 2.2 fold and DFR (Unigene100192_All) with 2.8 fold. The expression levels of the remaining down-regulated unigenes were in between 1 to 2 fold down-regulated.

### Further analysis on COG clusters

**Figure 8:**
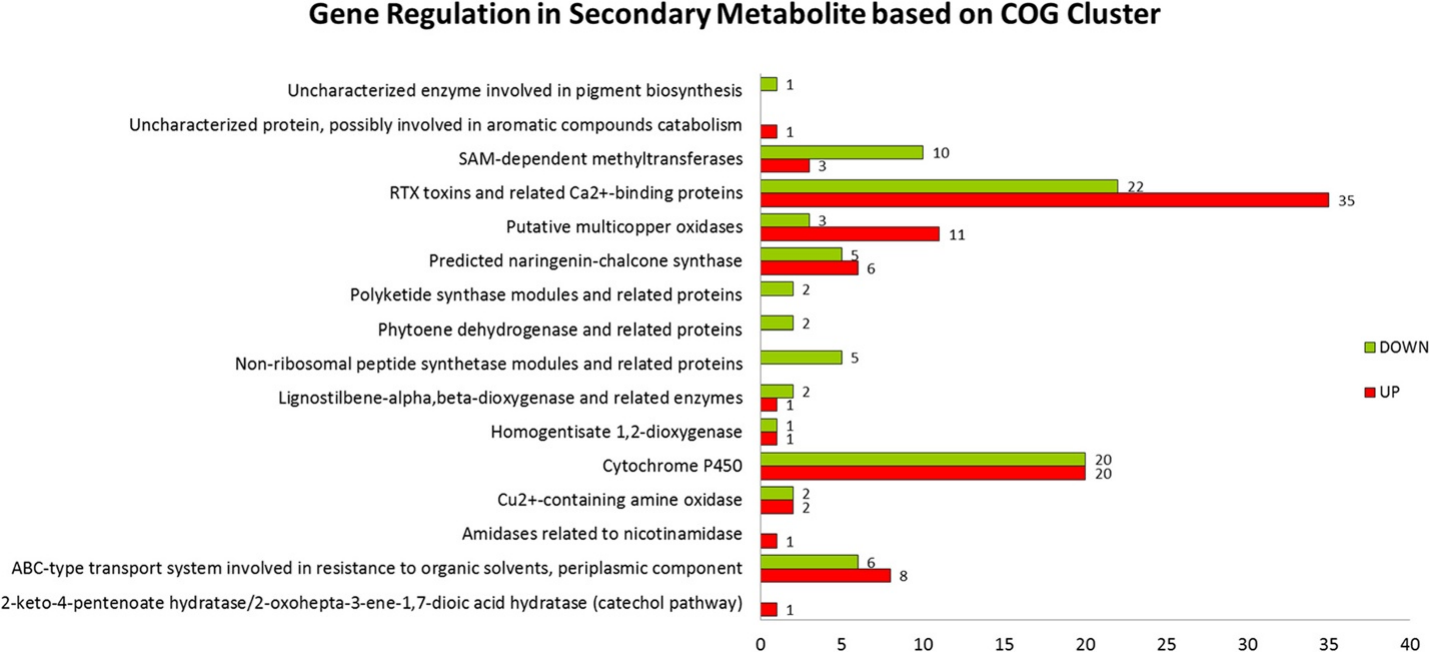
**Gene regulation in secondary metabolite cluster based on COG database classification.**

**Figure 9:**
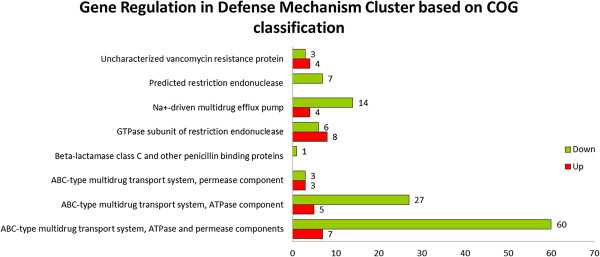
**Gene regulation in defence mechanism cluster based on COG database classification.**

Further analysis on the secondary metabolite cluster (Figure 8) and defense mechanism cluster (Figure 9) that was classified using the COG database, was carried out. Out of 16,426 unigenes that were assigned through the COG database, 171 unigenes were clustered in the secondary metabolite cluster with 90 up-regulated and 81 down-regulated unigenes respectively. In the secondary metabolite cluster, the highest number of up-regulated unigenes were RTX toxin and and related Ca2+ binding proteins with 35 unigenes, followed by cytochrome P450 (20 unigenes) and putative multicopper oxidases (11 unigenes). While the highest number of down-regulated unigenes were RTX toxin and and related Ca2+ binding proteins with 22 unigenes, followed by cytochrome P450 (20 unigenes) and SAM-dependent methyltransferases (10 unigenes).

In total, there were 152 unigenes that were mapped into the defense mechanism cluster in the COG database, with 31 and 121 up-regulated and down-regulated unigenes respectively. In the defense mechanism cluster, the highest down-regulated unigenes were ABC-type multidrug system, ATPase and permease components with 60 unigenes and ABC-type multidrug system, ATPase components with 27 unigenes.

### Experimental validation

**Figure 10:**
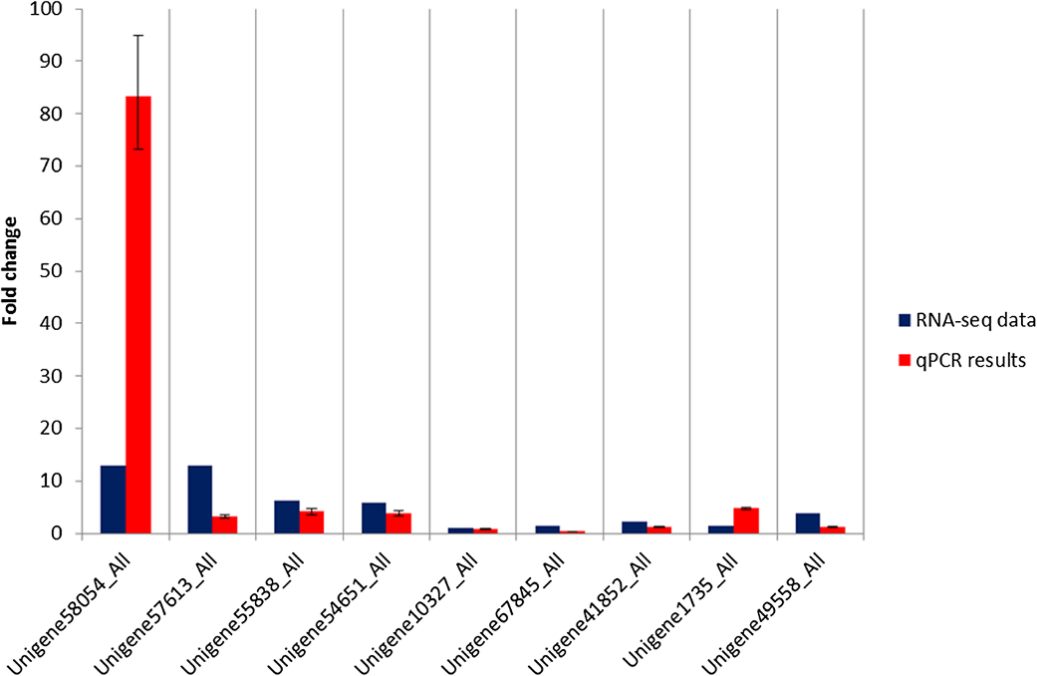
**Expression pattern validation of selected unigenes by qPCR.** Changes in transcript levels of 9 selected unigenes. X-axis shows –fold changes in transcript abundance of unigenes. Blue bar indicates transcript abundance changes calculated by the RPKM method. Red bar with associated standard error bar represents relative expression level determined by qPCR using 2^-∆∆CT^ method. Results represent mean standard deviations (±SD) of four experimental replicates.

The qPCR results of 9 randomly selected unigenes showed general agreement with their transcript abundance changes as determined by RNA-seq, suggesting the reliability of the transcriptome profiling data (Figure 10). For the unigenes tested only two showed some discrepancies although both were similarly up-regulated i.e Unigene58054_All showed a much higher expression level while Unigene1735_All had moderately higher expression in qPCR as compared to the RNA-seq results.

## Discussion

Digital gene expression (DGE) analysis for elucidating differentially expressed genes (DEGs) is an approach that can be used to further understand the nature of a plant’s response towards various stimuli or stresses [41]. For medicinal plants, RNA-Seq together with DEGs data has been used to identify genes that are directly or indirectly involved in the biosynthetic pathways of target bioactive compounds. Thus far, cytochrome P450 (CYP450) had been identified in most reported analysis of late terpenoid pathways. The combination of RNA-Seq technology and methyl jasmonate induction experiments successfully identified one CYP450 and four glycosyltransferases as key enzymes in the ginsenoside biosynthesis in *Panax quinquefolius*[42]. Subsequently, by combining RNA-Seq technology and phylogenetic tree analysis based on the previously identified CYP450 and glycosyltransferase in *Panax quinquefolius*, two CYP450 and one UDP-glycosyltransferase were also elucidated as candidates for ginsenoside biosynthesis in *Panax notoginseng*[42, 43]. Additionally, seven CYP450 and five glucosyltransferase were identified in mogrosides biosynthesis in *Siraitia grosvenorri*; and six CYP450 and one glucosidase identified in camptothecin biosynthesis in *Camtotheca acuminate*[44, 45]. Additionally, RNAseq analysis from different rhizomes of cultivated *Curcuma longa* cultivars in India described transcripts potentially related to anticancer and antimalarial terpenoids [46].

This strategy was also used to elucidate the candidate genes that might be involved in the panduratin A biosynthesis through sequencing of the whole transcriptome of untreated and phenylalanine treated cell suspension cultures of *Boesenbergia rotunda* and analysis of the gene expression patterns involved in the phenylpropanoid and flavonoid pathways. In total, there were 24, 473, 594 and 23, 470, 648 reads that were successfully generated from control and phenylalanine treated *Boesenbergia rotunda* respectively using an Illumina-Solexa sequencer (Table 1). Using SOAPdenovo software, these unigenes were overlapped and assembled to form longer sequences from short reads to contigs, scaffolds and unigenes, which resulted in 101, 043 *Boesenbergia rotunda* unigenes successfully assembled. However, only 50.41% unigenes were successfully annotated in the public protein databases with 49.93% in NR, followed by 34.63% in Swiss-Prot, 24.07% in KEGG and 16.26% in COG (Table 2). The limited numbers of identified plant genes and their deposition in the database might be the reason for the unannotated 49.59% of the transcriptome unigenes. These unannotated unigenes should be further identified to enrich public plant databases.

In future, further study using mutant yeast complementation experiments will be conducted to determine the unigene functions. This complementary experimental approach is based on employing the specific mutant yeast that cannot grow on certain media composition. However, mutant yeast harbouring the gene of interest could grow on the selectable media due to their ability to restore the physical deficiency of the mutated yeast [47–50], in which consequently indirectly verify their gene function. The functional complementary approach using mutant yeast has successfully elucidated genes in *Arabidopsis thaliana* such as isopenthyl diphosphate isomerase encoded by the IPP gene, methyltransferase encoded by the *COQ3* gene, phosphor-ethanilamine N-methyltransferase and acetylornithine aminotransferase encoded by the *TUP5* gene [47–50]. The same approach has also elucidated iron transporter gene function (*MxIRT1*) in *Malus xiaojinesis*[51].

Phenylalanine was chosen as an exogenous precursor to increase panduratin A production in *Boesenbergia rotunda* cell suspension culture at fourteen-days post treatment with phenylalanine [35]. The strategy was based on increasing the key compound within the phenylpropanoid biosynthetic pathway that would induce or increase the yield of the final product. The addition of phenylalanine was also reported to stimulate taxol production in *Taxus cupidata* cell suspension culture [52, 53].

Differential expression patterns of transcriptome profile between control and phenylalanine treated *Boesenbergia rotunda* cell suspension culture showed 14, 644 significantly up-regulated and 14, 379 significantly down-regulated unigenes with FDR ≤ 0.001 and |log_2_Ratio| ≥1 (Figure 3). The key factor of differential gene expression in *Boesenbergia rotunda* cell suspension culture between control and phenylalanine treated is proposed to be related to the differential expression of transcription factors and transcription regulators. Transcription factors are regulatory proteins that control the expression of specific groups of genes through sequence-specific DNA binding and protein-protein interactions. They act either as activators or repressors of gene expression, mediating either an increase or decrease in the accumulation of mRNA depending on tissue type or in response to internal or external signals [54, 55]. Both transcription factors and transcription regulators in the phenylalanine treated samples were initially regulated by the addition of phenylalanine and appear to result in regulation of other genes.

There were in total 139 transcription factors and 46 transcription regulators found in *Boesenbergia rotunda* in this study. The classification of both transcription factors and regulators were carried out by homology BlastX search against the rice database using iTAK software. The most abundant transcription factor found was classified under the C3H family. The second most abundant transcription factor found was MYB followed by NAC, WRKY, bZIP and AP2-EREBP family (Table 4). All of these transcription factor families except for CH3 showed significant differential expression of their members in the treated sample (FDR ≤ 0.001 and |log_2_Ratio| ≥1), suggesting that they played an important role in the induction or repression of the panduratin A biosynthesis pathway.

It was reported that plant R2R3 MYB transcription factor contain two helix-turn-helix motifs responsible for binding to target genes [56]. The R2R3 MYB family plays a major role in regulating sets of genes that are responsible for secondary metabolite biosynthetic pathways in plants especially for synthesizing flavonoids in the phenylpropanoid pathway [54]. Similarly it was apparent that MYB transcription factors were affected in this study. Although only one MYB gene was up-regulated, the remaining four were down-regulated and were possibly responsible for down regulating or switching off sets of genes that were not related to the panduratin A biosynthetic pathway. Other up-regulated transcription factors such as AP2-EREBP, WRKY, bZIP, GRAS and NAC haves been reported to modulate the genes for plant growth and plant response to biotic or abiotic stresses [57–61]. This information would be useful for future analysis on genes that are regulated by these transcription factors especially in relation to phenylpropanoid and flavonoid pathways.

The addition of exogenous phenylalanine in the liquid media of treated cell suspension culture helps to elucidate genes that might be directly or indirectly responsible for panduratin A biosynthesis. The most abundant up-regulated unigenes in the phenylpropanoid pathway were peroxidase, with 40 out of 90 unigenes (Table 8). Peroxidase is classified as a class III plant peroxidase that catalyzes plant-specific oxidoreduction between hydrogen peroxide (H_2_O_2_) and various reductants [62]. Differential expression profile of the peroxidase as isoenzymes in *Boesenbergia rotunda* suggests that they might be involved in catalyzing different substrate and may be involved in different physiological processes. Peroxidase class III is involved in lignification in higher plants by radical coupling of monolignols [63]. This oxidoreduction reaction utilizes hydrogen peroxide (H_2_O_2_) for oxidative power to produce monolignol radicals for lignin polymerization [63]. Different monolignols in this reaction produces different types of lignin and thus provide different resistance barriers for plants. Lignin provides mechanical strength and resistance against pathogens in plants. Lignification is a normal process for plant growth and development and also occurs in response to environmental stresses [64]. Additionally, some peroxidase isoenzymes were regulated upon environmental stimuli or prior attack by pathogens, which render the plant with self-defense mechanism against physical, chemical and biological stresses [65].

Most primary metabolic processes such as carbohydrate metabolism, energy metabolism and amino acid metabolism have more down-regulated unigenes compared to up-regulated unigenes in the same pathway (FDR ≤ 0.001 and |log_2_Ratio| ≥1) (Table 7). Primary metabolism is essential for plant growth, plant development and plant reproduction. In cell suspension cultures, primary metabolism is essential for plant cells to propagate in liquid media. Down-regulation of unigenes in the primary metabolic pathways after 14 days of propagation might be due to depleted nutrients in the liquid media. It can be suggested that by depleting plant nutrients, cell suspension cultures are stressed and eventually induces secondary metabolites. Similar observations were reported by Lattanzio *et. al*. who showed that under limited nutrient conditions, increased phenolic compounds was observed with a decrease in biomass production [66].

There are several hypotheses that relates the carbon limiting step in primary metabolism to secondary metabolite production in plants as a trade-off between growth and the production of carbon-based secondary metabolites such as phenolic compounds [67, 68]. The carbon-nutrient hypothesis (CNBH) suggests that plants modify the allocation of carbon skeletons between primary and secondary metabolism, where in a nutrient depletion situation, the plant restricts growth and the carbon skeleton is allocated to produce phenolic secondary metabolite compounds [68]. In addition, the protein competition model of phenolic allocation by Jones and Hartley, 1999 suggests that protein and phenolic synthesis are competing for the use of phenylalanine as a precursor [69]. Therefore, the availability of phenylalanine for phenolic compound biosynthesis is affected by any environmental changes that affect plant growth and protein synthesis.

In order to validate the transcriptome data, qPCR validation was done using a random selection of unigenes and included some of the unigenes that were annotated from the flavonoid pathway. This included PAL; Unigen10327_All, C4H; Unigene67845_All, 4CL; Unigene41852_All, CHS; Unigene1735_All and F3H; Unigene49558_All (Figure 10).

Figure 7 shows the proposed panduratin A biosynthetic pathway, which is derived from chalcones in the flavonoid pathway. Through RNA-Seq and differentially expressed genes analysis, genes that are potentially involved in panduratin A synthesis were identified (Tables 8 and 9). From the results, it can be inferred that the isoflavanoids biosynthetic pathway may not be present in *Boesenbergia rotunda* as the pathway map was not found in the KEGG results. Additionally, there was no unigene mapped to flavone synthase (FS), suggesting that flavones were not produced in the *Boesenbergia rotunda* cell suspension culture. However, in contrast , flavones were successfully isolated and identified in black rhizome of *Boesenbergia pandurata*[70, 71]. Tuchinda *et. al*. (2002), reported that *Boesenbergia pandurata* which is *Boesenbergia rotunda*’s synonym has four rhizome varieties including yellow, black, white and red rhizomes [23]. From the transcriptome data, it could be suggested that different rhizomes varieties may have different flavonoid biosynthesis pathways as the source of cell suspension culture in this study originated from yellow rhizomes and this would merit further investigation. The other enzyme that had no unigene mapped to it was anthocyanine reductase (ANR), which converts anthocyanidins to flavan-3-ols, which eventually polymerizes to form proanthocyanidins.

The other enzymes in the flavonoid pathway consist of both up- and down-regulated unigenes except for chalcone isomerase (CHI), dihydroflavonol-4-reductase (DFR) and leucoanthocyanidin reductase (LAR) (Figure 7). There were no significant gene regulation patterns in CHI, whereas down-regulated unigenes were identified for both DFR and LAR (Table 9). Most of unigenes that were mapped to the remaining flavonoid enzymes such as phenylalanine ammonia-lyase (PAL), cinnamate-4-hydroxylase (C4H), 4-coumaroyl:coenzyme A ligase (4CL), chalcone synthase (CHS), flavanone-3-hydroxylase (F3H), flavonol synthase (FLS) and anthocyanin synthase (ANS) were down-regulated. It is suggested that down-regulation of enzymes isomers in the flavonoid pathway causes switch-off of competitive pathways and eventually divertion of the metabolic flux to the production of the desired secondary metabolites.

There were three enzymes known to be directly involved in panduratin A production, PAL, 4CL and CHS (Figure 7) [72]. All of these enzymes are known to be encoded by a multi-gene family. Phenylalanine ammonia-lyase (PAL) catalyzes the first step in phenylpropanoid biosynthetic pathway. In many plant species, several copies of the *PAL* gene have been found and characterized. Between 2 to 4 *PAL* genes have been identified in Arabidopsis, tobacco, bean and parsley [73–76]. More than 40 *PAL* genes were identified in potato [77]. Although more than one *PAL* gene is present in each plant species, the regulation of each *PAL* gene depends on different response of stimuli [73]. In this study, there were 14 unigenes that were mapped as PAL. However, only 2 unigenes were up-regulated in response to the addition of phenylalanine.

A second enzyme 4CL, showed a gene regulation pattern directly involved in the panduratin A production. There were in total 44 unigenes mapped as 4CL in *Boesenbergia rotunda*. However, only 3 unigenes were up-regulated and 15 unigenes down-regulated after 14 days post treatment with phenylalanine. 4CL can be divided into two types in *Arabidopsis thaliana*; type I is responsible for lignin formation and type II leads to branching of the flavonoid pathways to produce flower pigments and defence mechanisms [78]. However, in rice, other than type I 4CL cluster, none were clustered in type II, but instead clustered separately in type III [79]. Although type I 4CL in dicots and type III 4CL in monocots are suggested to lead to lignin formation, they differ in sequences and substrate preference [79]. Similarly for type II 4CL, which also have differences in substrate preference and eventually causes branching in flavonoid biosynthetic pathway [78]. Hence it could be suggested that the remaining non-regulated 4CL in *Boesenbergia rotunda* might also be involved in lignin formation or possess different substrates preference.

Chalcone synthase (CHS) is categorized under the type III polyketide synthases superfamily [40]. It catalyzes the formation of chalcones by condensing one p-coumaroyl-CoA and three malonyl-CoA [80]. Different combination of thioesters and three malonyl-CoA were catalysed by CHS and eventually produce different chalcones for instance, a condensation reaction of p-coumaroyl-CoA gives rise to naringenin chalcone while condensation of cinnamoyl-CoA gives rise to pinocembrin chalcone [40]. It was reported that each CHS has a different substrate preference by *in vitro* determining CHS relative activity percentage [81]. Although more than one *CHS* gene was isolated from one species, some CHS isoenzymes were constitutively expressed throughout the plant development with varying expression levels but some were expressed upon induction by environmental stresses including wounding, UV light and pathogen infections [82]. In the transcriptome data, it was showed that one out of 25 CHS mapped in the KEGG database, Unigene1735_All, was up-regulated and seven other unigenes were down-regulated. Thus, it could speculate that Unigene1735_All is a key enzyme that directs the production of panduratin A. Comparison of Unigene1735_All with other CHS in the NR database, the homology search results showed that Unigene1735_All was highly similar to 3-ketoacyl synthase with 53-57% similarity. Functional studies of Unigene1735_All could be carried out to determine the substrate specificity and their derivative products.

From the findings, it was showed that some of the unigenes that were mapped to PAL, CHS, F3H and ANS were up-regulated. In maize, the C1 MYB transcription factor regulates PAL, CHS, F3H, DFR, ANS and UDP-glucose-flavonol glucosyltransferase in the flavonoid pathway [54]. In the results, one MYB transcription factor was up-regulated (Table 4) and correlated with the results of up-regulated unigenes with the exception of DFR. Hence it is possible that the up-regulated MYB in the *Boesembergia rotunda* transcriptome data is the key regulator for up-regulating this set of unigenes, with the exception of DFR, in the flavonoid pathway. It is suggested that MYB could be a potential target for strategies to overproduce panduratin A in *Boesenbergia rotunda*.

Gene regulation patterns in the flavonoid pathway (Table 10) shows that the expression levels of unigenes for anthocyanin production such as F3H, FLS and ANS were higher compared to chalcone production at 1.8 – 3.8 fold higher in phenylalanine-treated samples than the control. In contrast, higher fold down-regulation was observed for chalcone production which includes PAL, 4CL and CHS showing a range 1.8 – 3.3 fold lower compared to the control. Therefore, it could be inferred that exogenous phenylalanine induction causes a metabolic pathway shift towards higher anthocyanin production, and indirectly increases panduratin A production. Unigenes that may be indirectly involved in the panduratin A biosynthetic pathway are shown in Table 10. Genes directly involved remain to be elucidated until a reference pathway is available. Nevertheless, the unknown unigenes may be involved and this merits further studies such as complementary experiment approach and gene overexpression studies as strategies to understand the unknown pathway.

## Conclusion

This is the first report of *Boesenbergia rotunda* transcriptome data to elucidate gene regulation pathways in response to exogenous phenylalanine treatment. Through RNA-Seq and differentially expressed genes (DEGs) analysis, gene regulation patterns in the panduratin A biosynthetic pathway was analysed in particular with respect to the flavonoid pathway. Although enzymes that are directly involved in panduratin A production through chalcone remains to be elucidated, other unigenes appear as promising targets for strategies of overproduction of panduratin A in *Boesenbergia rotunda* through a metabolic engineering strategy. The transcriptome data will also enrich the plant database as a reference for other Zingiberceae family members.

## Methods

### Plant material

The cell suspension cultures were initiated from meristems of the yellow rhizome variety of *Boesenbergia rotunda* obtained through the Plant Biotechnology Research Laboratory, University of Malaya and were from plants grown under natural conditions. The suspension cultures were propagated in Murashige and Skoog (MS) liquid media [83] supplemented with 1 mg/l of 6-benzylaminopurine (BAP), 1 mg/l of napthtalene acetic acid (NAA), 1 mg/l of biotin, 2 mg/L of 2,4-dichlorophenoxyacetic acid and 99.42 mg/l of L-glutamine and cultured according to the method described in Tan *et. al*. 2012 [35]. The cultures were propagated in 250 ml conical flasks shaken at 70-80 rpm using an orbital shaker at 25 ± 2°C under a 16 h photoperiod with a light intensity of 31.4 μmol/m^2^/s provided by cool fluorescent lamp in the growth room.

Equal amounts of the cell suspension (5 ml of settled cell volume) were used in all experiments. For the control, no phenylalanine was added, while for phenylalanine-treated samples, 40 mg/l of phenylalanine was added into the propagation media at the beginning of the experiment.

### Total RNA extraction

Phenylalanine-treated cell cultures were harvested after 14 days of propagation. The liquid media was removed and the samples were deep frozen in liquid nitrogen. A modified cetylmethylammonium bromide (CTAB) method was employed to extract total RNA from both control and phenylalanine-treated cell suspension cultures [37]. Initially, 300 to 500 milligram of cell suspension culture was ground in liquid nitrogen. Subsequently the ground sample was added into 2 ml microcentrifuge tubes containing 1 ml of pre-heated CTAB extraction buffer with 20 μl *β*-mercaptoethanol. Then, the tube was heated at 65°C for 10 minutes. The mixture was vortexed for few seconds to mix it well. An equal volume of chloroform:isoamyl alcohol (24:1; v/v) was then added to the mixture and the mixture vortexed for few seconds.

The mixture of DNA and lysed debris cells was centrifuged in an Eppendorf 5417R centrifuge (Eppendorf, Hamburg, Germany) at 10 621 X g for 15 minutes to remove protein impurities. The supernatant was recovered and transferred into a new microcentrifuge tube and the steps repeated 2-3 times. Next, 0.1 volumes of 3 M sodium acetate together with 3 volumes of pre-cooled absolute ethanol were added to the supernatant. The mixture was kept at -80°C for 2–3 days to precipitate RNA and then centrifuged at 10 621 X g for 30 minutes at 4°C. The supernatant was discarded and the remaining pellet washed with 1 ml of cold 70% (v/v) ethanol. The sample was again centrifuged at 10 621 X g for 5 minutes at 4°C, removed and pellet was air-dried and dissolved in 20 μl DEPC-treated water.

### Library preparation and sequencing

The quality and quantity of RNA samples were analysed using an Agilent 2100 Bioanalyzer (Agilent, Waldbronn, Germany) to ensure RNA concentrations of more than 400 ng/μl and to obtain RNA quality with an OD 260/280 of between 1.8 – 2.2, 28S/18S > 1.8 and an RNA integrity number (RIN) ≥ 8. Whole transcriptome sequencing was carried out using an Illumina-Solexa (Illumina Inc, San Diego, CA, USA) platform at Beijing Genome Institute (BGI), Shenzhen, China.

The Illumina-Solexa platform sequences short fragments of genomic RNA by employing sequence-by synthesis technology. Total RNA samples are sheared by nebulization to yield short fragments approximately between 200–700 bp. Then, cDNA fragments were synthesized by priming these short RNA fragments using random hexamer. Subsequently, two different adaptors were ligated at both ends of the fragments. Single stranded cDNA fragments were then randomly bound on the inside surface of the flow cell channels. Next, the fragments are amplified by solid-phase bridge amplification method. After several PCR cycles, several million dense clusters of double stranded DNA are generated in each channel of the flow cells. Finally, high-throughput sequencing was performed using Illumina-Solexa sequence analyser.

### Transcript assembly and annotation

Sequence data generated from the Illumina-Solexa sequencer was transformed by base calling into sequence data, called raw data or raw reads. The raw data generated from Solexa was filtered by removing the 3′ adaptor. Then, the clean data were assembled into transcript contigs by short reads assembling program SOAPdenovo software [84]. This software adopts de the Bruijn graph data sequence data structure to construct contigs. The read were mapped back to the contigs and using the paired-end relationship between reads, contigs from the same transcript can be detected. Next, scaffolds were made by connecting the contigs using SOAPdenovo, in which N represents the unknown sequence between each two contigs. Paired-end reads were used again to fill the intra-scaffold gaps to form unigenes. As two samples, which were treated and control from the sample species were sequenced, unigenes from each sample’s assembly were further assembled to acquire longer non-redundant unigenes using TGI clustering tools [85].

Longer unigenes that were generated by combining both transcripts from control and treated samples were annotated against protein databases such as NR, Swiss-Prot, KEGG and COG by Blastx (e-value cutoff of < 0.00001) alignment. The best aligned results were used to determine the sequence direction of the unigenes. Next, for other unaligned unigenes, sequence orientation as well as its coding regions was predicted by using ESTscan software [39].

### Unigene functional annotation and expression level

Functional annotation was done to show the unigenes’ protein functional information, which includes protein orthologous groups and pathway annotation. All unigenes with sequence orientation was subjected to functional annotation. Homology search was done by Blastx alignment of unigenes against public protein databases such as NR, Swiss-Prot, KEGG (Kyoto Encyclopedia of Genes and Genomes) and COG (Clusters of Orthologous Groups) with e-value < 0.00001. Next, in order to classify unigenes in Gene Ontologous (GO) functional annotation, unigenes with NR annotation information was mapped to their respective ontologies using Blast2GO program [86] and further gene classification was done using WEGO software [87]. Unigenes were classified under three GO-terms namely molecular function, cellular component and biological process. The level of transcripts or unigenes was determined using Reads per kb per Million reads (RPKM) method [88].

### Digital Gene Expression analysis for elucidating differentially expressed genes (DEGs)

In order to identify genes that have different expression levels between control and phenylalanine-treated samples, analysis of differentially expressed genes (DEGs) was done by employing Poisson distribution calculations [89].

### Identification and classification of transcription factors and transcription regulators

Transcription factors (TFs) and transcription regulators (TRs) in *Boesenbergia rotunda* was identified and classified using iTAK software. iTAK is a program to identify and classify plant transcription factors (TFs) and transcription regulators (TRs) from protein or nucleotide sequences based on the rules (required and forbidden protein domains of each gene family) described in [90]. Protein sequences that were translated from nucleotide sequences generated from the Illumina sequencer were used to find both transcription factors and transcription regulators. iTAK searches both TFs and TRs based on homology search using TFs and TRs from rice database. Subsequently, the differentially regulated TFs and TRs in response to phenylalanine were also identified.

### KEGG pathway enrichment analysis

Different genes usually cooperate with each other to exercise their biological functions. Pathway-based analysis helps to further understand genes biological functions. Kyoto Encyclopedia of Genes and Genomes (KEGG) is the major public pathway-related database. Pathway enrichment analysis identifies significantly enriched metabolic pathways or signal transduction pathways in DEGs comparing with the whole genome background.

### Validation and expression pattern analysis

To experimentally validate the transcriptional abundance results from sequencing and computational analysis, 9 unigenes were selected for qPCR analysis. The unigenes include 4 random up-regulated unigenes (Unigene58054_All, Unigene57613_All, Unigene555838_All and Unigene54651_All) and 5 unigenes that are annotated in the flavonoid pathway (Unigene10327_All; PAL, Unigene67845_All; C4H, Unigene41852_All; 4CL, Unigene1735_All; CHS and Unigene49558_All; F3H). Primers that were used for the experimental validation are shown in Additional file 7. The dissociation curves for all target unigenes are shown in Additional file 8. Reverse transcription reactions were performed using *TransScript*®II Reverse Transcriptase (TransgenBiotech, Beijing, China) with approximately 2 μg total RNA following the manufacturer’s instructions. Primers for qPCR were designed using Primer 3 software. Elongation factor was used as the reference gene. qPCR was performed on QuantStudio 12 K Flex realtime PCR platform (Applied Biosystem, Carlsbad, CA, USA) using Power SYBR® Green Master Mix (Applied Biosystem, Carlsbad, CA, USA) to detect transcript abundance. The amplification was achieved by the following PCR protocol: first denaturation at 95°C for 10 minutes, then 40 cycles of denaturation at 95°C for 15 s, annealing and extension at 60°C for 1 minute. The dissociation curve was established at the end of PCR cycle at 95°C for 15 s, 60°C for 1 minute followed by 95°C for 15 s. The relative expression levels of the selected unigenes normalized to elongation factor was calculated using 2^-∆∆Ct^ method. All reactions were performed with four experimental replicates and data were analyzed using QuantStudio 12 K Flex software.

### Availability of supporting data

The RNA-seq data supporting the results of this article are available at the NCBI under BioProject with accession number PRJNA256116 with SRA Study accession number SRR1524841 for control untreated and SRR1524842 for phenylalanine treated samples.

## Electronic supplementary material

Additional file 1: **The length distribution of control unigene, phenylalanine treated unigene and All Unigene.** All Unigene is a long sequence unigene that is derived from combining both control and phenylalanine treated unigene. (PDF 36 KB)

Additional file 2: **The gap distribution of control unigene, phenylalanine treated unigene and All Unigene.** All Unigene is a long sequence unigene that is derived from combining both control and phenylalanine treated unigene. (PDF 36 KB)

Additional file 3: **This table summarizes the number of unigenes that have been assigned in COG functional categories.** (PDF 42 KB)

Additional file 4: **Unigene that are assigned to GO-terms which is classified under biological process, cellular components and molecular function.** (PDF 43 KB)

Additional file 5: **KEGG phenylpropanoid pathway containing gene expression patterns.** Red borders represent enzyme that consist of up-regulated unigenes while green borders represent enzyme consist of down-regulated unigenes. Both up- and down-regulated unigenes that mapped to the same enzyme were marked as both red and green borders. (PNG 19 KB)

Additional file 6: **KEGG flavonoid pathway containing gene expression patterns.** Red borders represent enzymes that consist of up-regulated unigenes while green borders represent enzymes consisting of down-regulated unigenes. Both up- and down-regulated unigenes that mapped to the same enzyme were marked as both red and green borders. (PNG 22 KB)

Additional file 7: **Primers used for experimental validation.** (PDF 31 KB)

Additional file 8: **Dissociation curves of target unigenes in qPCR.** (PNG 936 KB)

## Abbreviations

NR: non-redundant
KEGG: Kyoto Encyclopedia of Genes and Genomes
COG: Cluster Orthologous Group (COG)
PAL: phenylalanine ammonia-lyase
C4H: cinnamic-4-hydroxylase
4CL: 4-coumaroyl:coenzyme A ligase
CHS: chalcone synthase
CHI: chalcone isomerase
FS1/FS2: flavone synthase 1 and 2
IFS: isoflavone synthase
DFR: dihydroflavonol 4-reductase
F3H: flavanone-3-hydroxylase
FLS: flavonol synthase
ANS: anthocyanidin synthase
LAR: leucoanthocyanidin reductase
ANR: anthocyanidin reductase
MS: Murashige and Skoog
BAP: 6-benzylaminopurine
NAA: napthtalene acetic acid
CTAB: cetyltrimethylammonium bromide
MGI: Malaysia Genome Institute
BGI: Beijing Genome Institute
RIN: RNA integrity number
PCR: polymerase chain reaction
cDNA: complementary deoxyribonucleic acid
DNA: deoxyribonucleic acid
RNA: ribonucleic acid
SOAP: Short Oligonucleotide Analysis Package
RPKM: Reads per kb per Million reads
TFs: Transcription factors
TRs: Transcription regulators
DEGs: Differentially expressed genes
FDR: False Discovery Rate
DGE: Digital gene expression.

## References

[1] Perry LM, Metzger J (1980). Medicinal Plants of East and Southeast Asia: Attributed Properties and Uses.

[2] Larsen K (1996). Preliminary checklist of the Zingiberaceae of Thailand. Thai Forest Bull (Bot).

[3] Burkill IH, Birtwistle W, Foxworthy FW, Scrivenor JB, Watson JG (1966). A dictionary of the Economic Products of the Malay Peninsula.

[4] Saralamp P, Chuakul W, Temsiririrkkul R, Clayton T (1996). Medicinal Plants in Thailand.

[5] Ching AYL, Tang SW, Sukari MA, Lian GEC, Rahmani M, Khalid K (2007). Characterization of flavonoid derivatives from *Boesenbergia rotunda* (L.). Malays J Ann Sci.

[6] Wijayakusuma HMH (2001). Tumbuhan Berkhasiat Obat Indonesia: Rempah, Rimpang Dan Umbi.

[7] Jaipetch T, Kanghae S, Pancharoen O, Patrick V, Reutrakul V, Tuntiwachwuttikul P, White A (1982). Constituents of *Boesenbergia pandurata* (syn. *Kaempferia pandurata*): isolation, crystal structure and synthesis of (±)-Boesenbergin A. Aust J Chem.

[8] Mongkolsuk S, Dean FM (1964). Pinostrobin and alpinetin from *Kaempferia pandurata*. J Chem Soc.

[9] Morikawa T, Funakoshi K, Ninomiya K, Yasuda D, Miyagawa K, Matsuda H, Yoshikawa M (2008). Medicinal foodstuffs. XXXIV. Structures of new prenylchalcones and prenylflavanones with TNF-alpha and aminopeptidase N inhibitory activities from Boesenbergia rotunda. Chem Pharm Bull.

[10] Trakoontivakorn G, Nakahara K, Shinmoto H, Takenaka M, Onishi-Kameyama M, Ono H, Yoshida M, Nagata T, Tsushida T (2001). Structural analysis of a novel antimutagenic compound, 4-hydroxypanduratin A, and the antimutagenic activity of flavonoids in a Thai spice, fingerroot (*Boesenbergia pandurata* Schult.) against mutagenic heterocyclic amines. J Agric Food Chem.

[11] Tuntiwachwuttikul P, Pancharoen O, Reutrakul V, Byrne L (1984). (1′RS,2′SR,6′RS)-(2,6-Dihydroxy-4-methoxyphenyl)-[3′-methyl-2′-(3″-methylbut-2″-enyl)-6′-phenyl-cyclohex-3′-enyl] methanone (panduratin A)-a constituent of the red rhizomers of a variety of *Boesenbergia pandurata*. Aust J Chem.

[12] Hwang JK, Chung JY, Baek NI, Park JH (2004). Isopanduratin A from *Kaempferia pandurata* as an active antibacterial agent against cariogenic *Streptococcus mutans*. Int J Antimicrob Agents.

[13] Kiat TS, Pippen R, Yusof R, Ibrahim H, Khalid N, Rahman NA (2006). Inhibitory activity of cyclohexenyl chalcone derivatives and flavonoids of fingerroot, *Boesenbergia rotunda* (L.), towards dengue-2 virus NS3 protease. Bioorg Med Chem Lett.

[14] Yun JM, Kwon H, Mukhtar H, Hwang JK (2005). Induction of apoptosis by panduratin A isolated from *Kaempferia pandurata* in human colon cancer HT-29 cells. Planta Med.

[15] Yun JM, Kweon MH, Kwon H, Hwang JK, Mukhtar H (2006). Induction of apoptosis and cell cycle arrest by a chalcone panduratin A isolated from *Kaempferia pandurata* in androgen-independent human prostate cancer cells PC3 and DU145. Carcinogenesis.

[16] Kirana C, Jones GP, Record IR, McIntosh GH (2007). Anticancer properties of panduratin A isolated from *Boesenbergia pandurata* (Zingiberaceae). J Nat Med.

[17] Win NN, Awale S, Esumi H, Tezuka Y, Kadota S (2007). Bioactive secondary metabolites from *Boesenbergia pandurata* of Myanmar and their preferential cytotoxicity against human pancreatic cancer PANC-1 cell line in nutrient-deprived medium. J Nat Prod.

[18] Cheah S-C, Appleton DR, Lee S-T, Lam M-L, Hadi AHA, Mustafa MR (2011). Panduratin A inhibits the growth of A549 cells through induction of apoptosis and inhibition of NF-KappaB translocation. Molecules.

[19] Yanti OHI, Anggakusuma, Hwang JK (2009). Effects of panduratin A isolated from *Kaempferia pandurata* Roxb. on the expression of matrix metalloproteinase-9 by *Porphyromonas gingivalis* supernatant-induced KB cells. Biol Pharm Bull.

[20] Yanti A, Gwon SH, Hwang JK (2009). *Kaempferia pandurata* Roxb. inhibits *Porphyromonas gingivalis* supernatant-induced matrix metalloproteinase-9 expression via signal transduction in human oral epidermoid cells. J Ethnopharmacol.

[21] Tewtrakul S, Subhadhirasakul S, Karalai C, Ponglimanont C, Cheenpracha S (2009). Anti-inflammatory effects of compounds from *Kaempferia parviflora* and *Boesenbergia pandurata*. Food Chem.

[22] Yun JM, Kwon H, Hwang JK (2003). *In vitro* anti-inflammatory activity of panduratin A isolated from *Kaempferia pandurata* in RAW264. 7 cells. Planta Med.

[23] Tuchinda P, Reutrakul V, Claeson P, Pongprayoon U, Sematong T, Santisuk T, Taylor WC (2002). Anti-inflammatory cyclohexenyl chalcone derivatives in *Boesenbergia pandurata*. Phytochemistry.

[24] Cheenpracha S, Karalai C, Ponglimanont C, Subhadhirasakul S, Tewtrakul S (2006). Anti-HIV-1 protease activity of compounds from *Boesenbergia pandurata*. Bioorg Med Chem.

[25] Rukayadi Y, Lee KH, Hwang JK, Yanti (2009). Activity of panduratin A isolated from *Kaempferia pandurata* Roxb. against multi-species oral biofilms *in vitro*. J Oral Sci.

[26] Rukayadi Y, Lee K, Han S, Yong D, Hwang JK (2009). *In vitro* activities of panduratin A against clinical *Staphylococcus* strains. Antimicrob Agents Chemother.

[27] Shim JS, Kwon YY, Han YS, Hwang JK (2008). Inhibitory effect of panduratin A on UV-induced activation of mitogen-activated protein kinases (MAPKs) in dermal fibroblast cells. Planta Med.

[28] Shim JS, Kwon YY, Hwang JK (2008). The effects of panduratin A isolated from *Kaempferia pandurata* on the expression of matrix metalloproteinase-1 and type-1 procollagen in human skin fibroblasts. Planta Med.

[29] Sohn JH, Han KL, Lee SH, Hwang JK (2005). Protective effects of panduratin A against oxidative damage of tert-butylhydroperoxide in human HepG2 cells. Biol Pharm Bull.

[30] Shindo K, Kato M, Kinoshita A, Kobayashi A, Koike Y (2006). Analysis of antioxidant activities contained in the *Boesenbergia pandurata* Schult. rhizome. Biosci Biotechnol Biochem.

[31] Kim D-Y, Kim M-S, Sa B-K, Kim M-B, Hwang J-K (2012). *Boesenbergia pandurata* attenuates diet-induced obesity by activating AMP-activated protein kinase and regulating lipid metabolism. Int J Mol Sci.

[32] WHO (2014). Dengue and Severe Dengue. Factsheet No11.

[33] WHO (2014). Dengue Vaccine Research.

[34] Chee CF, Abdullah I, Buckle MJC, Rahman NA (2010). An efficient synthesis of (±)-panduratin A and (±)-isopanduratin A, inhibitors of dengue-2 viral activity. Tetrahedron Lett.

[35] Tan EC, Karsani SA, Foo GT, Wong SM, Abdul Rahman N, Khalid N, Othman S, Yusof R (2012). Proteomic analysis of cell suspension cultures of *Boesenbergia rotunda* induced by phenylalanine: identification of proteins involved in flavonoid and phenylpropanoid biosynthesis pathways. Plant Cell Tiss Org.

[36] Herrmann KM, Weaver LM (1999). The shikimate pathway. Annu Rev Plant Biol.

[37] Al-Obaidi JR, Mohd-Yusuf Y, Chin-Chong T, Mhd-Noh N, Othman RY (2010). Identification of a partial oil palm polygalacturonase-inhibiting protein (EgPGIP) gene and its expression during basal stem rot infection caused by *Ganoderma boninense*. Afr J Biotechnol.

[38] Li R, Yu C, Li Y, Lam TW, Yiu SM, Kristiansen K, Wang J (2009). SOAP2: an improved ultrafast tool for short read alignment. Bioinformatics.

[39] Iseli C, Jongeneel CV, Bucher P (1999). ESTScan: a program for detecting, evaluating, and reconstructing potential coding regions in EST sequences. Proc Int Conf Intell Syst Mol Biol.

[40] Austin MB, Noel JP (2003). The chalcone synthase superfamily of type III polyketide synthases. Nat Prod Rep.

[41] Molina C, Zaman-Allah M, Khan F, Fatnassi N, Horres R, Rotter B, Steinhauer D, Amenc L, Drevon JJ, Winter P (2011). The salt-responsive transcriptome of chickpea roots and nodules via deepSuperSAGE. BMC Plant Biol.

[42] Sun C, Li Y, Wu Q, Luo H, Sun Y, Song J, Lui EMK, Chen S (2010). *De novo* sequencing and analysis of the American ginseng root transcriptome using a GS FLX Titanium platform to discover putative genes involved in ginsenoside biosynthesis. BMC Genomics.

[43] Luo H, Sun C, Sun Y, Wu Q, Li Y, Song J, Niu Y, Cheng X, Xu H, Li C (2011). Analysis of the transcriptome of *Panax notoginseng* root uncovers putative triterpene saponin-biosynthetic genes and genetic markers. BMC Genomics.

[44] Sun Y, Luo H, Li Y, Sun C, Song J, Niu Y, Zhu Y, Dong L, Lv A, Tramontano E (2011). Pyrosequencing of the *Camptotheca acuminata* transcriptome reveals putative genes involved in camptothecin biosynthesis and transport. BMC Genomics.

[45] Tang Q, Ma X, Mo C, Wilson IW, Song C, Zhao H, Yang Y, Fu W, Qiu D (2011). An efficient approach to finding *Siraitia grosvenorii* triterpene biosynthetic genes by RNA-seq and digital gene expression analysis. BMC Genomics.

[46] Annadurai RS, Neethiraj R, Jayakumar V, Damodaran AC, Rao SN, Katta MA, Gopinathan S, Sarma SP, Senthilkumar V, Niranjan V (2013). *De novo* transcriptome assembly (NGS) of *Curcuma longa* L. rhizome reveals novel transcripts related to anticancer and antimalarial terpenoids. PLoS One.

[47] Campbell M, Hahn FM, Poulter CD, Leustek T (1998). Analysis of the isopentenyl diphosphate isomerase gene family from *Arabidopsis thaliana*. Plant Mol Biol.

[48] Avelange-Macherel M-H, Joyard J (1998). Cloning and functional expression of *AtCOQ3*, the *Arabidopsis* homologue of the yeast *COQ3* gene, encoding a methyltransferase from plant mitochondria involved in ubiquinone biosynthesis. Plant J.

[49] Bolognese CP, McGraw P (2000). The isolation and characterization in yeast of a gene for arabidopsis S-adenosylmethionine: phospho-ethanolamine *N*-methyltransferase. Plant Physiol.

[50] Frémont N, Riefler M, Stolz A, Schmülling T (2013). The Arabidopsis *TUMOR PRONE5* Gene Encodes an acetylornithine aminotransferase required for arginine biosynthesis and root meristem maintenance in blue light. Plant Physiol.

[51] Zhang X-N, Han Z-H, Yin L-L, Kong J, Xu X-F, Zhang X-Z, Wang Y (2013). Heterologous functional analysis of the *Malus xiaojinensis MxIRT1* gene and the His-box motif by expression in yeast. Mol Biol Rep.

[52] Fett-Neto AG, Melanson SJ, Sakata K, DiCosmo F (1993). Improved growth and taxol yield in developing calli of *Taxus cuspidata* by medium composition modification. Nat Biotechnol.

[53] Fett-Neto AG, Melanson SJ, Nicholson SA, Pennington JJ, DiCosmo F (1994). Improved taxol yield by aromatic carboxylic acid and amino acid feeding to cell cultures of *Taxus cuspidata*. Biotechnol Bioeng.

[54] Vom Endt D, Kijne JW, Memelink J (2002). Transcription factors controlling plant secondary metabolism: what regulates the regulators?. Phytochemistry.

[55] Broun P (2004). Transcription factors as tools for metabolic engineering in plants. Curr Opin Plant Biol.

[56] Du H, Zhang L, Liu L, Tang XF, Yang WJ, Wu YM, Huang YB, Tang YX (2009). Biochemical and molecular characterization of plant MYB transcription factor family. Biochemistry (Mosc).

[57] Mizoi J, Shinozaki K, Yamaguchi-Shinozaki K (2012). AP2/ERF family transcription factors in plant abiotic stress responses. Biochim Biophys Acta.

[58] Ülker B, Somssich IE (2004). WRKY transcription factors: from DNA binding towards biological function. Curr Opin Plant Biol.

[59] Jakoby M, Weisshaar B, Dröge-Laser W, Vicente-Carbajosa J, Tiedemann J, Kroj T, Parcy F (2002). bZIP transcription factors in Arabidopsis. Trends Plant Sci.

[60] Hirsch S, Oldroyd GED (2009). GRAS-domain transcription factors that regulate plant development. Plant Signal Behav.

[61] Olsen AN, Ernst HA, Leggio LL, Skriver K (2005). NAC transcription factors: structurally distinct, functionally diverse. Trends Plant Sci.

[62] Hiraga S, Sasaki K, Ito H, Ohashi Y, Matsui H (2001). A large family of class III plant peroxidases. Plant Cell Physiol.

[63] Marjamaa K, Kukkola EM, Fagerstedt KV (2009). The role of xylem class III peroxidases in lignification. J Exp Bot.

[64] Kim YH, Kim CY, Song WK, Park DS, Kwon SY, Lee HS, Bang JW, Kwak SS (2008). Overexpression of sweetpotato swpa4 peroxidase results in increased hydrogen peroxide production and enhances stress tolerance in tobacco. Planta.

[65] Hiraga S, Yamamoto K, Ito H, Sasaki K, Matsui H, Honma M, Nagamura Y, Sasaki T, Ohashi Y (2000). Diverse expression profiles of 21 rice peroxidase genes. FEBS Lett.

[66] Lattanzio V, Cardinali A, Ruta C, Fortunato IM, Lattanzio VM, Linsalata V, Cicco N (2009). Relationship of secondary metabolism to growth in oregano (*Origanum vulgare* L.) shoot cultures under nutritional stress. Environ Exp Bot.

[67] Herms DA, Mattson WJ (1992). The dilemma of plants: to grow or defend. Q Rev Biol.

[68] Lerdau M, Coley PD (2002). Benefits of the carbon-nutrient balance hypothesis. Oikos.

[69] Jones CG, Hartley SE (1999). A protein competition model of phenolic allocation. Oikos.

[70] Jaipetch T, Reutrakul V, Tuntiwachwuttikul P, Santisuk T (1983). Flavonoids in the black rhizomes of *Boesenbergia panduta*. Phytochemistry.

[71] Herunsalee A, Pancharoen O, Tuntiwachwuttikul P (1987). Further studies of flavonoids of the black rhizomes *Boesenbergia pandurata*. J Sci Soc Thailand.

[72] Bowsher C, Steer MW, Tobin AK (2008). Plant biochemistry.

[73] Lois R, Dietrich A, Hahlbrock K, Schulz W (1989). A phenylalanine ammonia-lyase gene from parsley: structure, regulation and identification of elicitor and light responsive cis-acting elements. EMBO J.

[74] Wanner LA, Li G, Ware D, Somssich IE, Davis KR (1995). The phenylalanine ammonia-lyase gene family in *Arabidopsis thaliana*. Plant Mol Biol.

[75] Fukasawa-Akada T, Kung SD, Watson J (1996). Phenylalanine ammonia-lyase gene structure, expression, and evolution in *Nicotiana*. Plant Mol Biol.

[76] Cramer C, Edwards K, Dron M, Liang X, Dildine S, Bolwell GP, Dixon R, Lamb C, Schuch W (1989). Phenylalanine ammonia-lyase gene organization and structure. Plant Mol Biol.

[77] Joos HJ, Hahlbrock K (2005). Phenylalanine ammonia-lyase in potato (*Solanum tuberosum* L.). Eur J Biochem.

[78] Ehlting J, Büttner D, Wang Q, Douglas CJ, Somssich IE, Kombrink E (2002). Three 4-coumarate: coenzyme A ligases in *Arabidopsis thaliana* represent two evolutionarily divergent classes in angiosperms. Plant J.

[79] Gui J, Shen J, Li L (2011). Functional characterization of evolutionarily divergent 4-coumarate: Coenzyme A ligases in rice. Plant Physiol.

[80] Ferrer JL, Jez JM, Bowman ME, Dixon RA, Noel JP (1999). Structure of chalcone synthase and the molecular basis of plant polyketide biosynthesis. Nat Struct Biol.

[81] Yamazaki Y, Suh DY, Sitthithaworn W, Ishiguro K, Kobayashi Y, Shibuya M, Ebizuka Y, Sankawa U (2001). Diverse chalcone synthase superfamily enzymes from the most primitive vascular plant, *Psilotum nudum*. Planta.

[82] Wingender R, Röhrig H, Höricke C, Wing D, Schell J (1989). Differential regulation of soybean chalcone synthase genes in plant defence, symbiosis and upon environmental stimuli. Mol Gen Genet.

[83] Murashige T, Skoog F (1962). A revised medium for rapid growth and bio assays with tobacco tissue cultures. Physiol Plant.

[84] Li R, Zhu H, Ruan J, Qian W, Fang X, Shi Z, Li Y, Li S, Shan G, Kristiansen K (2010). *De novo* assembly of human genomes with massively parallel short read sequencing. Genome Res.

[85] Pertea G, Huang X, Liang F, Antonescu V, Sultana R, Karamycheva S, Lee Y, White J, Cheung F, Parvizi B (2003). TIGR Gene Indices clustering tools (TGICL): a software system for fast clustering of large EST datasets. Bioinformatics.

[86] Conesa A, Götz S, García-Gómez JM, Terol J, Talón M, Robles M (2005). Blast2GO: a universal tool for annotation, visualization and analysis in functional genomics research. Bioinformatics.

[87] Ye J, Fang L, Zheng H, Zhang Y, Chen J, Zhang Z, Wang J, Li S, Li R, Bolund L (2006). WEGO: a web tool for plotting GO annotations. Nucleic Acids Res.

[88] Mortazavi A, Williams BA, McCue K, Schaeffer L, Wold B (2008). Mapping and quantifying mammalian transcriptomes by RNA-Seq. Nat Methods.

[89] Audic S, Claverie J-M (1997). The significance of digital gene expression profiles. Genome Res.

[90] Pérez-Rodríguez P, Riaño-Pachón DM, Corrêa LGG, Rensing SA, Kersten B, Mueller-Roeber B (2010). PlnTFDB: updated content and new features of the plant transcription factor database. Nucleic Acids Res.

