# Supplementary material for: Transcriptome profiling shows gene regulation patterns in a flavonoid pathway in response to exogenous phenylalanine in Boesenbergia rotunda cell culture
Source: BMC Genomics. 2014 Nov 18;15(1):984. doi: 10.1186/1471-2164-15-984 (PMC4289260; doi:10.1186/1471-2164-15-984)
Supplement: Supplementary file 2 — Additional file 2: The gap distribution of control unigene, phenylalanine treated unigene and All Unigene. All Unigene is a long sequence unigene that is derived from combining both control and phenylalanine treated unigene. (PDF 36 KB) [file 12864_2013_6859_MOESM2_ESM.pdf]

Additional file 2: The gap distribution of control unigene, phenylalanine treated unigene and All Unigene. All Unigene is longer sequence unigene that derived from combining both control and phenylalanine treated unigene.

	Control Unigene		Phenylalanine treated Unigene		All Unigene	
Gap Percentage (%)	Number of Unigene	Percentage (%)	Number of Unigene	Percentage (%)	Number of Unigene	Percentage (%)
0~5	67,308	85.20	68,919	88.88	83,678	82.81
5~20	9,207	11.65	7,186	9.27	14,019	13.87
20~40	2,478	3.14	1,434	1.85	3,340	3.31
40~60	5	0.01	2	0.00	6	0.01
60~80	0	0.00	0	0.00	0	0.00
80~100	0	0.00	0	0.00	0	0.00
Total	78,998		77,541		101,043	
