# Supplementary material for: Transcriptome profiling shows gene regulation patterns in a flavonoid pathway in response to exogenous phenylalanine in Boesenbergia rotunda cell culture
Source: BMC Genomics. 2014 Nov 18;15(1):984. doi: 10.1186/1471-2164-15-984 (PMC4289260; doi:10.1186/1471-2164-15-984)
Supplement: Supplementary file 3 — Additional file 3: This table summarizes the number of unigenes that have been assigned in COG functional categories. (PDF 42 KB) [file 12864_2013_6859_MOESM3_ESM.pdf]

**Additional file 3: This table summarized the number of unigenes that assigned in COG functional categories.**

| Code | Functional-Categories | Gene-Number |
|---|---|---|
| A | RNA processing and modification | 276 |
| B | Chromatin structure and dynamics | 277 |
| C | Energy production and conversion | 882 |
| D | Cell cycle control, cell division, chromosome partitioning | 1908 |
| E | Amino acid transport and metabolism | 1217 |
| F | Nucleotide transport and metabolism | 334 |
| G | Carbohydrate transport and metabolism | 1943 |
| H | Coenzyme transport and metabolism | 534 |
| I | Lipid transport and metabolism | 839 |
| J | Translation, ribosomal structure and biogenesis | 2171 |
| K | Transcription | 3691 |
| L | Replication, recombination and repair | 3053 |
| M | Cell wall/membrane/envelope biogenesis | 1503 |
| N | Cell motility | 240 |
| O | Posttranslational modification, protein turnover, chaperones | 2614 |
| P | Inorganic ion transport and metabolism | 826 |
| Q | Secondary metabolites biosynthesis, transport and catabolism | 753 |
| R | General function prediction only | 4851 |
| S | Function unknown | 1863 |
| T | Signal transduction mechanisms | 2630 |
| U | Intracellular trafficking, secretion, and vesicular transport | 1018 |
| V | Defense mechanisms | 395 |
| W | Extracellular structures | 4 |
| Y | Nuclear structure | 12 |
| Z | Cytoskeleton | 600 |
| Total | | 34434 |
