# Supplementary material for: Transcriptome profiling shows gene regulation patterns in a flavonoid pathway in response to exogenous phenylalanine in Boesenbergia rotunda cell culture
Source: BMC Genomics. 2014 Nov 18;15(1):984. doi: 10.1186/1471-2164-15-984 (PMC4289260; doi:10.1186/1471-2164-15-984)
Supplement: Supplementary file 4 — Additional file 4: Unigene that are assigned to GO-terms which is classified under biological process, cellular components and molecular function. (PDF 43 KB) [file 12864_2013_6859_MOESM4_ESM.pdf]

Additional file 4: Unigene that assigned to GO-terms which classified under biological process, cellular component and molecular function.

Ontology	GO-term	Number of Unigene
Biological Process (10,040 Unigenes)	anatomical structure formation	91
	biological adhesion	3
	biological regulation	734
	cell killing	0
	cellular component biogenesis	121
	cellular component organization	204
	cellular process	3012
	death	23
	developmental process	109
	establishment of localization	624
	growth	18
	immune system process	9
	localization	627
	locomotion	0
	metabolic process	3032
	multi-organism process	79
	multicellular organismal process	122
	pigmentation	697
	reproduction	48
	reproductive process	44
	response to stimulus	443
	rhythmic process	0
	viral reproduction	0
Cellular Component (16,493 Unigenes)	cell	5448
	cell part	5448
	envelope	125
	extracellular region	81
	extracellular region part	5
	macromolecular complex	587
	membrane-enclosed lumen	82
	organelle	4158
	organelle part	551
	symplast	0
	synapse	0
	synapse part	0
	virion	4
	virion part	4

Molecular Function (7,451 Unigenes)	antioxidant	36
	auxiliary transport protein	0
	binding	3302
	catalytic	2880
	chemoattractant	0
	chemorepellent	0
	electron carrier	137
	enzyme regulator	35
	metallochaperone	0
	molecular transducer	108
	nutrient reservoir	6
	proteasome regulator	0
	protein tag	0
	structural molecule	188
	transcription regulator	274
	translation regulator	60
	transporter	425
