# Supplementary figures and images for: Transcriptome profiling shows gene regulation patterns in a flavonoid pathway in response to exogenous phenylalanine in Boesenbergia rotunda cell culture
Source: BMC Genomics. 2014 Nov 18;15(1):984. doi: 10.1186/1471-2164-15-984 (PMC4289260; doi:10.1186/1471-2164-15-984)

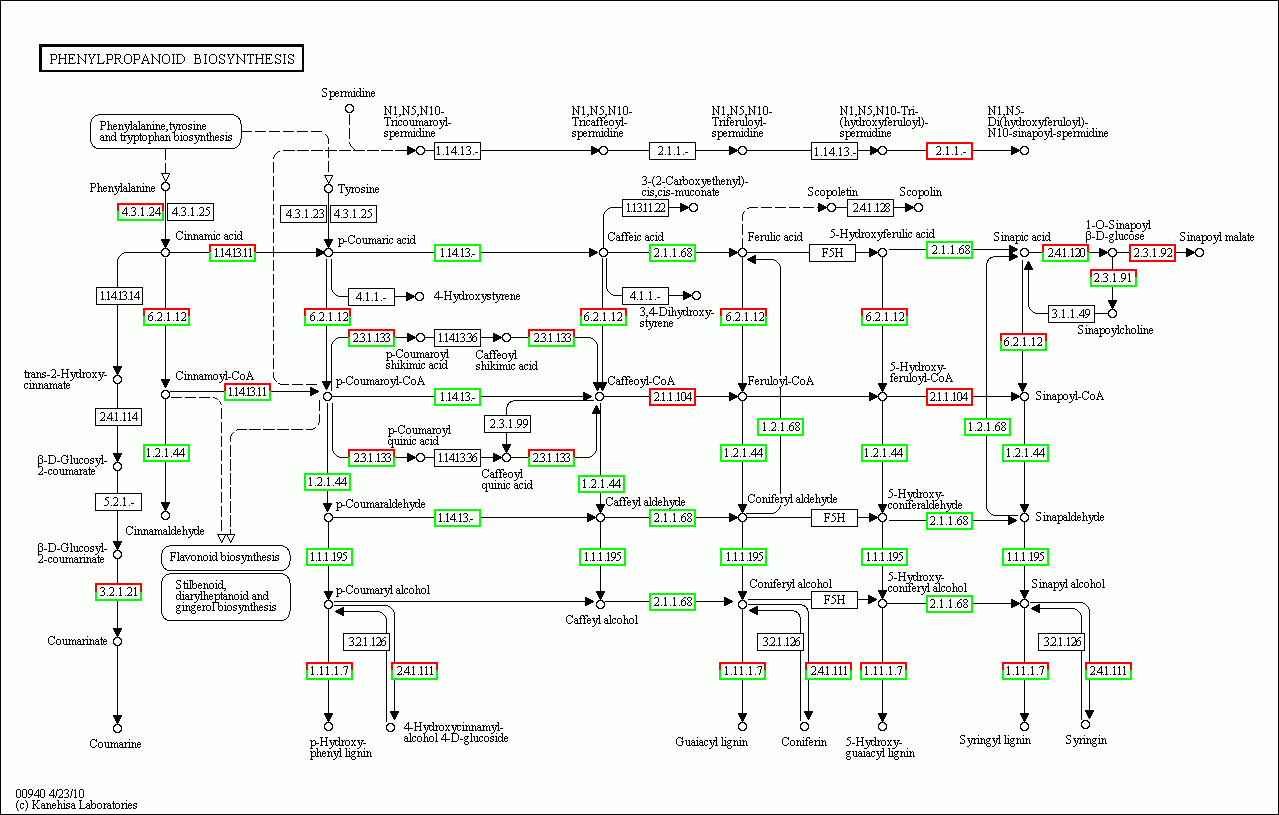

Supplement: Supplementary file 5 — Additional file 5: KEGG phenylpropanoid pathway containing gene expression patterns. Red borders represent enzyme that consist of up-regulated unigenes while green borders represent enzyme consist of down-regulated unigenes. Both up- and down-regulated unigenes that mapped to the same enzyme were marked as both red and green borders. (PNG 19 KB) [file 12864_2013_6859_MOESM5_ESM.png]

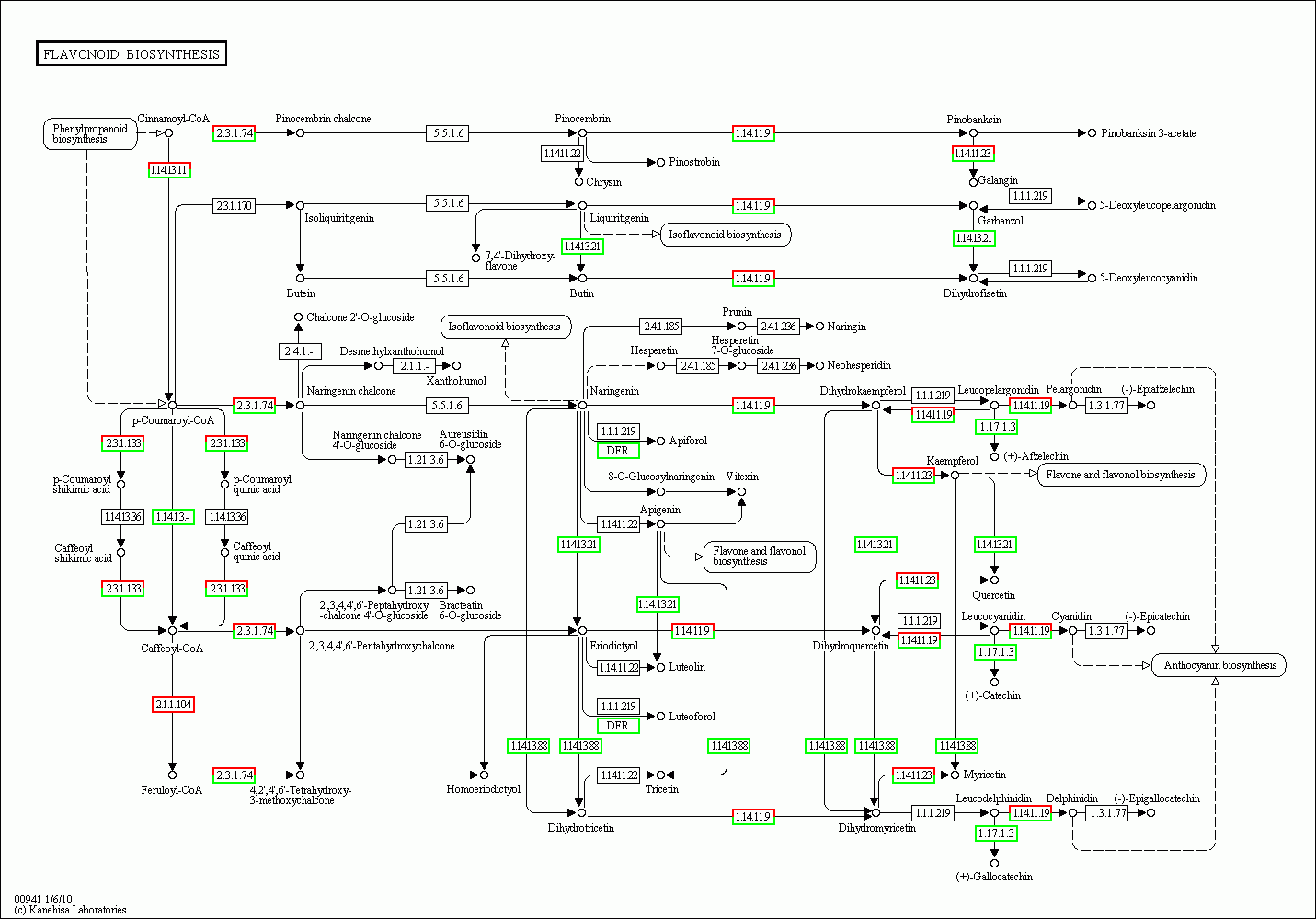

Supplement: Supplementary file 6 — Additional file 6: KEGG flavonoid pathway containing gene expression patterns. Red borders represent enzymes that consist of up-regulated unigenes while green borders represent enzymes consisting of down-regulated unigenes. Both up- and down-regulated unigenes that mapped to the same enzyme were marked as both red and green borders. (PNG 22 KB) [file 12864_2013_6859_MOESM6_ESM.png]

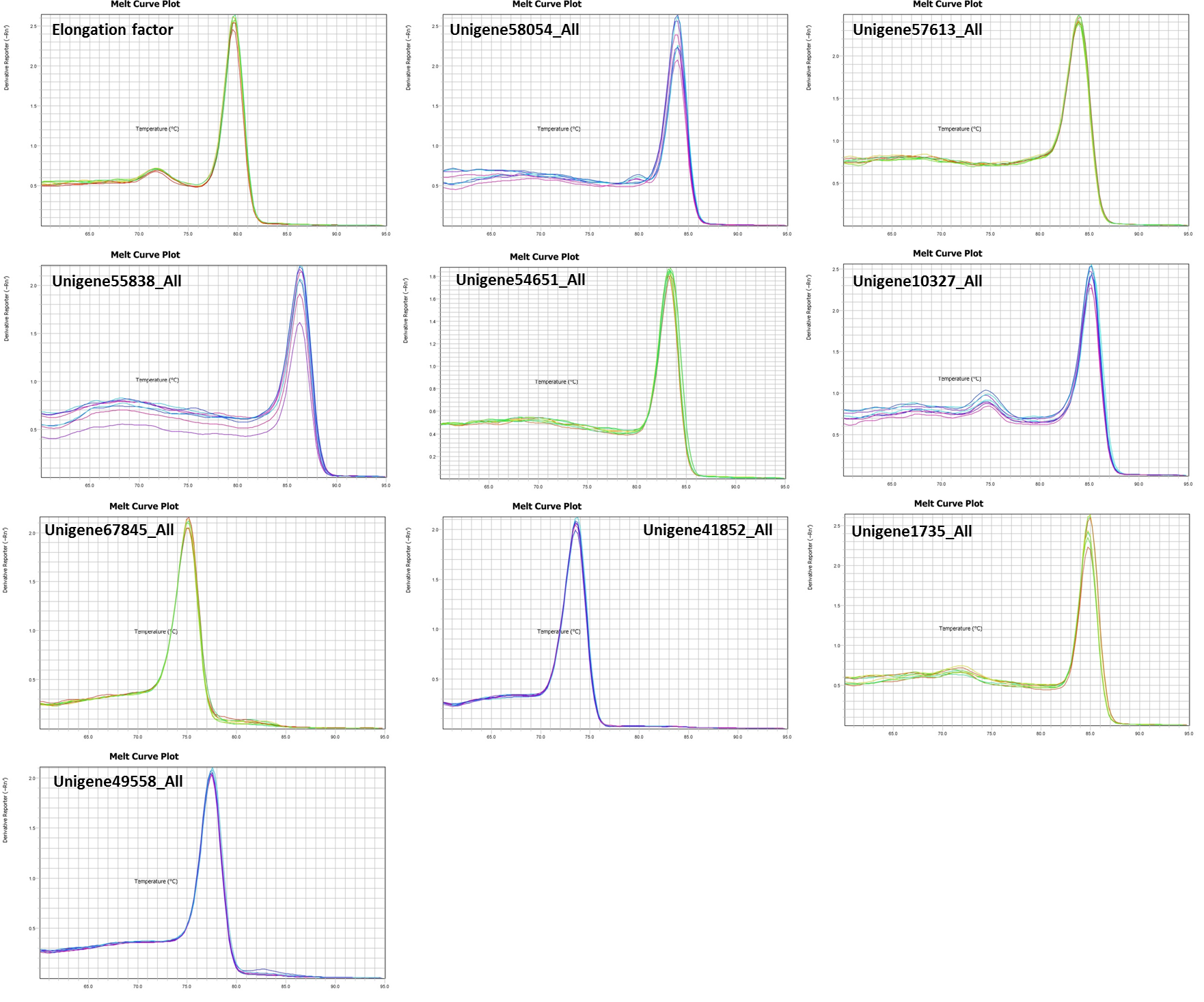

Supplement: Supplementary file 8 — Additional file 8: Dissociation curves of target unigenes in qPCR. (PNG 936 KB) [file 12864_2013_6859_MOESM8_ESM.png]
