# Supplementary material for: Transcriptome profiling shows gene regulation patterns in a flavonoid pathway in response to exogenous phenylalanine in Boesenbergia rotunda cell culture
Source: BMC Genomics. 2014 Nov 18;15(1):984. doi: 10.1186/1471-2164-15-984 (PMC4289260; doi:10.1186/1471-2164-15-984)
Supplement: Supplementary file 7 — Additional file 7: Primers used for experimental validation. (PDF 31 KB) [file 12864_2013_6859_MOESM7_ESM.pdf]

Additional file 7: Primers used for experimental validation.

>Unigene58054_All Forward
ACAACGCCTTCAACAACCTC

> Unigene58054_ All Reverse
CTGGTGGTCTGGAATGTGGT

> Unigene57613_ All Forward
CGAGCTGTATGGGGAAGAAG

> Unigene57613_ All Reverse
GCCGCGTAGGAGATGAAGTA

> Unigene55838_ All Forward
GGTGGTGTACCAGGTTCAGG

> Unigene55838_ All Reverse
GACGCCAGAGATCGTGACAG

> Unigene54651_ All Forward
ATAAACAGCCAGGGCAACAG

> Unigene54651_ All Reverse
TTCAGCATCAGATCCCCTTC

> Unigene10327_ All Forward
GCTCTGCAAAATGGACCTCA

> Unigene10327_ All Reverse
ATCGAGCAGTTGGTGTCCTC

> Unigene67845_ All Forward
ATTCCTGTTTTGGGGCTTTC

> Unigene67845_ All Reverse
GGCATTTGTAGCGTTGCTTT

> Unigene41852_ All Forward
CCTAAATCTGCCTCAGGAAA

> Unigene41852_ All Reverse
GAGTTTACACTGAAGGCTTTTGC

> Unigene1735_ All Forward
GCTACAAGGCCAAGAAGACG

> Unigene1735_ All Reverse
CTCAGGGAGGTACGTGTCGT

> Unigene49558_ All Forward
ATAAGACGCTCTCCCGTTGT

> Unigene49558_ All Reverse
ATGGGTTCAGGCTTGACTCG
